# Supplementary material for: Revision of the Subgenus Ochthomantis Frogs from Madagascar (Amphibia: Mantellidae) with the Description of Four Species and Resurrection of Mantidactylus catalai and M. poissoni
Source: Animals (Basel). 2023 Sep 3;13(17):2800. doi: 10.3390/ani13172800 (PMC10563080; doi:10.3390/ani13172800)
Supplement: Supplementary file 1 [file animals-13-02800-s001.zip › Table S1_Voucher.pdf]

Table S1. Samples included in molecular analyses with locality. Samples are grouped according to species as determined by analyses presented here. Voucher specimen identification is provided for samples newly sequenced for this study and Genbank Accession numbers are provided for all samples.

Species	Voucher	Genbank Accession	Species	Voucher	Genbank Accession
M. peraccae	-	AY454391	*M. femoralis*	-	AY848151
M. cowanii	-	MK447691	*M. femoralis*	-	AY848138
M. lugubris	-	AY341710	*M. femoralis*		HQ610916
M. guttulatus	-	FJ559237	*M. femoralis*		HQ610913
M. ambony	RAN38009	OR466827	*M. femoralis*		HQ610914
M. ambony	-	AY324822	*M. macrotympanum* n. sp.	RAN39170	OR466806
M. ambony	-	HQ610870	*M. macrotympanum* n. sp.	RAN39125	OR466807
M. ambreensis	RAX6552	OR466843	*M. macrotympanum* n. sp.	RAX2715	OR466808
M. ambreensis	RAX9589	OR466844	*M. majori*	RAX8907	OR466809
M. ambreensis	RAN38501	OR466846	*M. mocquardi*	RAN42737	OR466835
	-	AY848129	*M. mocquardi*	RAX7524	OR466836
M. catalai	RAN44491	OR466824	*M. mocquardi*	RAX9022	OR466837
M. catalai	RAX10563	OR466825	*M. mocquardi*	RAX3669	OR466838
M. catalai	RAX10599	OR466826	*M. mocquardi*	RAN37992	OR466839
M. catalai	-	AY324821	*M. mocquardi*	RAX3641	OR466840
M. danieli n. sp.	RAN38186	OR466829	*M. mocquardi*	RAX4670	OR466841
M. danieli n. sp.	RAX10204	OR466830	*M. mocquardi*	RAX3915	OR466842
M. danieli n. sp.	RAX4268	OR466831	*M. mocquardi*	-	AF215317
M. danieli n. sp.	RAX10392	OR466832	*M. mocquardi*	-	HQ610921
M. danieli n. sp.	RAX6595	OR466833	*M. mocquardi*	-	JX570508
M. danieli n. sp.	RAN39387	OR466834	*M. mocquardi*	-	HQ610861
M. danieli n. sp.	-	AY324818	*M. mocquardi*	-	AY848150
M. danieli n. sp.	-	AY324816	*M. olgae* n. sp.	RAX3203	OR466821
M. femoralis	RAX2703	OR466810	*M. olgae* n. sp.	RAX5195	OR466822
M. femoralis	RAX2761	OR466811	*M. olgae* n. sp.	RAX10205	OR466823

M. femoralis	RAX6345	OR466812	*M. poissoni*	RAX9367	OR466847
M. femoralis	RAX8133	OR466813	*M. tavaratra* n. sp.	RAX11535	OR466848
M. femoralis	RAX7523	OR466814	*M. tavaratra* n. sp.	RAX11534	OR466849
M. femoralis	RAX9498	OR466815	*M. tavaratra* n. sp.	RAX11533	OR466850
M. femoralis	RAX7196	OR466816	*M. tavaratra* n. sp.	RAX11532	OR466851
M. femoralis	RAX10901	OR466817	*M. tavaratra* n. sp.	RAX10323	OR466852
M. femoralis	RAX10606	OR466818	*M. tavaratra* n. sp.	RAN39071	OR466853
M. femoralis	RAN35289	OR466819	*M. tavaratra* n. sp.	RAX6901	OR466854
M. femoralis	RAX8896	OR466820	*M. tavaratra* n. sp.	RAX5769	OR466855
M. femoralis	-	AY843698	*M. tavaratra* n. sp.	RAX6028	OR466856
M. femoralis	-	HQ610845	*M. tavaratra* n. sp.	RAX6107	OR466857
M. femoralis	-	HM364726	*M. tavaratra* n. sp.	RAX6838	OR466858
M. femoralis	-	HQ610917	*M. tavaratra* n. sp.	RAN43366	OR466859
M. femoralis	-	HQ610907	*M. tavaratra* n. sp.	RAX5310	OR466860
M. femoralis	-	HQ610911	*M. tavaratra* n. sp.	RAX5400	OR466861
M. femoralis	-	AY324815	*M. tavaratra* n. sp.	RAX5000	OR466828
M. femoralis	-	GU975183	*M. tavaratra* n. sp.	-	AY324819
M. femoralis	-	AY324817	*M. tavaratra* n. sp.	-	AY324820
M. femoralis	-	JF903917	*zolitschka*	-	HQ610866
M. femoralis	-	HQ610847	*zolitschka*	-	HQ610867
M. femoralis	-	HQ610918			

**Sample**	**Voucher catalog**	**Molecular ID**	**Locality**
RAX 2373	A167482	*M. ambreensis*	Betaindambo, Tsaratanana 530 m
RAX 6552	A167501	*M. ambreensis*	Irony River 970 m
RAX 9589	A174618	*M. ambreensis*	Ambohibola Forest, 300 m
RAN 38501	UMMZ 212435	*M. ambreensis*	Montagne d'Ambre Antomboka Fitsahana 650 m
RAN 38009	UMMZ 212426	*M. ambony*	Montagne d'Ambre Antomboka River 1150 m
RAX 7196	A174623	*M. femoralis*	Ankafina Forest 1420 m

RAX 7523	A174627	*M. femoralis*	Betampona Strict Nature Reserve 350 m
RAN 35289	UMMZ197651	*M. femoralis*	Manantantely forest 90 m
RAX 10606	A181735	*M. femoralis*	Beampingaratra Pass, Anosy Montane 520 m
RAX 8896	A174651	*M. femoralis*	Kianjavato-Vatovavy 150 m
RAX 2703	A167580	*M. femoralis*	Antsahatelo, Tsaratanana Reserve 800 m
RAX 2761	A167581	*M. femoralis*	Ramena 730 m
RAX 6345	A167521	*M. femoralis*	Bemanevika Lakes 1450 m
RAX 10955	UADBA	*M. femoralis*	Mandraka 1140 m
RAX 10901	A187128	*M. femoralis*	Itremo 1650 m
RAX 8133	A174646	*M. femoralis*	Manasamena 950 m
RAX 9498	A174654	*M. femoralis*	Ambohibevavy-Vasiana 850 m
RAN 42737	UMMZ 212881	*M. mocquardi*	Ankavanana river, Masoala 70-100 m
RAX 7524	A174628	*M. mocquardi*	Betampona Strict Nature Reserve 350 m
RAX 9022	A174652	*M. mocquardi*	Ambodiriana 100 m
RAN 37992	UMMZ 212824	*M. mocquardi*	Manantenina River, Marojejy Reserve 700 m
RAX 3641	UADBA 19647	*M. mocquardi*	Ambolokopatrika, Bataolana-Andapa 810 m
RAX 3669	A174621	*M. mocquardi*	Ambolokopatrika, Betaolana-Andapa 860 m
RAX 4670	A174622	*M. mocquardi*	Ankitsika, Vohemar 600 m
RAX 3915	A167587	*M. mocquardi*	Bezavona, Vohemar 530 m
RAX 9367	A174653	*M. poissoni*	Mandraka 1250 m
RAN 44491	UMMZ 212890	*M. catalai*	Iatara river, Andringitra 720 m
RAX 10563	A181732	*M. catalai*	Beampingaratra Pass, Anosy Montane 490 m
RAX 10599	A181821	*M. catalai*	Beampingaratra Pass, Anosy Montane 1140 m
RAX 4268	A167590	*M. danieli* n. sp.	Salafaina 400m
RAX 10204	A181773	*M. danieli* n. sp.	Andramanalana 840 m
RAN 38186	UMMZ 212827	*M. danieli* n. sp.	Antomboka river, Montagne d'Ambre 1150 m
RAN 39387	UMMZ 212836	*M. danieli* n. sp.	Antsahabe river, Manongarivo Reserve 180 m
RAX 10392	A181731	*M. danieli* n. sp.	Tsararano 490 m
RAX 6595	A167523	*M. danieli* n. sp.	Irony River 970 m
RAN 39071	UMMZ 212440	*M. tavaratra* n. sp.	Bekolosy, Manongarivo Reserve 1100 m
RAX 10323	A181730	*M. tavaratra* n. sp.	Andramanalana 1300m

RAX 5769	A167510	*M. tavaratra* n. sp.	Befosa, Tsaratanana 1680◎1700 m
RAX 6028	A167515	*M. tavaratra* n. sp.	Bemanevika Lakes 1600 m
RAX 6107	A167516	*M. tavaratra* n. sp.	Bemanevika Lakes 1600 m
RAX 6901	A167525	*M. tavaratra* n. sp.	Lohanandroranga 1440 m
RAX 6838	A167524	*M. tavaratra* n. sp.	Lohanandroranga 1650 m
RAN 43366	UMMZ 212889	*M. tavaratra* n. sp.	Befosa river, Tsaratanana 1630 m
RAX 5310	A167505	*M. tavaratra* n. sp.	Befosa, Tsaratanana 1600 m
RAX 5400	A167506	*M. tavaratra* n. sp.	Matsaborimaiky Lake, Tsaratanana Reserve 1950 m
RAX 11532	none	*M. tavaratra* n. sp.	Marojejy 1550 m
RAX 11533	none	*M. tavaratra* n. sp.	Marojejy 1550 m
RAX 11534	A187088	*M. tavaratra* n. sp.	Marojejy 1550 m
RAX 11535	A187089	*M. tavaratra* n. sp.	Marojejy 1550 m
RAX 5000	A167593	*M. tavaratra* n. sp.	Sorata 1300◎1350 m
RAX 3203	A167565	*M. olgai* n. sp.	Antsaravy Ridge, Tsaratanana Reseve 1150 m
RAX 5195	A167596	*M. olgai* n. sp.	Sorata 970 m
RAX 10205	A181726	*M. olgai* n. sp.	Andramanalana 850 m
RAN 39170	UMMZ 201416	*M. macrotympanum* n. sp.	Ambalafary, Manongarivo 250 m
RAX 2715	A167599	*M. macrotympanum* n. sp.	Antsahatelo, Tsaratanana Reserve 800 m
RAN 39125	UMMZ 213447	*M. macrotympanum* n. sp.	Ambalafary, Manongarivo 250 m
GENBANK	HQ610866 (ZSM 1768/2007)	*M. zolitschka*	An'ala Forest 840 m
GENBANK	HQ610867 (ZSM 1841/2007)	*M. zolitschka*	An'ala Forest 840 m
RAX 8907	A174620	*M. majori*	Kianjavato-Vatovavy 150 m
GENBANK	AY454391	*Spinomantis peraccae*	
GENBANK	MK447691	*Mantidactylus cowanii*	
GENBANK	AY341710	*Mantidactylus lugubris*	
GENBANK	FJ559237	*Mantidactylus guttulatus*	
