# Supplementary material for: Revision of the Subgenus Ochthomantis Frogs from Madagascar (Amphibia: Mantellidae) with the Description of Four Species and Resurrection of Mantidactylus catalai and M. poissoni
Source: Animals (Basel). 2023 Sep 3;13(17):2800. doi: 10.3390/ani13172800 (PMC10563080; doi:10.3390/ani13172800)
Supplement: Supplementary file 1 [file animals-13-02800-s001.zip › Table S13_distribution details.pdf]

Table S13. Species collecting localities. *: Specimens with molecular data

Species	Elevation range (m)	Observation sites	Geographic position	Specimens
Mantidactylus femoralis	350–1300	Ampanasana Ankolony–Beambihy river Marojejy	14°26.2'S 49°46.5'E	AMNH A157112, UADBA 7778
	700	Manantenina river Marojezy	14°26'S 49°46'E	UMMZ 212835, UADBA 3720
	1400	Analapakila	14°26.233'S 48°36.696'E	AMNH A167521*
	800	Antsahatelo	13°51.588'S 48°51.979'E	AMNH A 167580*
	750	Ramena	13°55.071'S 48°53.179'E	AMNH A 167581*
	1000	Les Rousettes	12°31'S 49°10'E	UADBA 9049, 9052, 9054, 9086–9087
	–	Moramanga	–	AMNH A23781, AMNH A50336
	420	Namarafana Zahamena	17°44'S 48°58.5'E	UADBA 26267
	850	Volontsagana Zahamena	17°42'S 48°46'E	UADBA 26375, 26379, 26399
	1150	Rangovalo Zahamena	17°40.5'S 48°45.5'E	UADBA 26378; UADBA 26253–26254, 26374, 26398
	250–450	Betampona Reserve	17°55.866'S 49°12.190'E	AMNH A174627*, UADBA 26250–26251, 26263–26265, 26402
	450	Sandranantitra	18°2.9'S 49°5.5'E	UADBA 12306, 12308
	950	Didy	18°11.858'S 48°34.728'E	UADBA 7278, 11886
	600–1100	Mangerivola	18°11.5' S 48°55.01'E	UADBA 19691, UADBA 19693–19694
	1 580	Ambohitantely	18°11.158'S 47°16.757'E	UADBA 26282; 26400, 39003
	730–950	Andranomangoboka	18°14.787'S 45°21.419'E	UADBA 26255, 26380
	1 200	Doany	18°17.297'S 45°33.289'E	UADBA 26281, 26376
	1 250	Andriankely	18°25.225'S 47°56.245'E	UADBA 20478–20479

1 300	Andranomay	18°28.8'S 47°57.3'E	UADBA 26363
850	Sahanomanana	18°32'S 48°26'E	UADBA 7515–7516
530	Andriatantely	18°41.724'S 48°48.769'E	UADBA 11974–11976
895	Sahaberiana	18°47.503'S 48°25.572'E	UADBA 11890
870–1050	Andranomanaponga	18°49.750'S 48°25.9'E	UADBA19663–19665
980	Analamay Ambatovy	18°49.715'S 48°19.960'E	UADBA 9251–9252, 9254
950	Mantadia Analamazaotra	18°56'S 48°24'E	UADBA 7241, 11887
1335	Mandraka	18°57.733'S 47°55.05'E	UADBA RAX10955*
950	Manasamena	19°02.637'S 48°20.910'E	AMNH A174646*, UADBA 26266, 26385
1025	Ampanatovana	19°02.637'S 48°20.912'E	UADBA 26118, 26268, 26269–26270
950–1000	Ankeniheny	19°05.850'S 48°19.850'E	UADBA 6280–6281
950	Tsinjoarivo	19°25' S 47°25' E	UADBA 8525, 8527, 9046, 26395–26396
850	Ambohibehivavy Vasiana	19°41.387' S 46°62.853' E	AMNH A174654*
1 600	Ambatomenaloha Itremo	20°37.130'S 46°33.347'E	AMNH A187128*
1000	Vohidratiana	21°0.117'S 47°33.500'E	UADBA 19696
1150–1420	Ankafina Tsarafidy	21°12.598'S 47°12.874'E	AMNH A174623*, UADBA 26249, 26262, 26389
1 210	Volontsagana river Andringitra	21°12.598'S 47°12.874'E	UADBA 26379, 26403
810	Sahavatoy River Andringitra	22°13.667'S 47°0.217'E	UADBA 26252

	150	Kianjavato	21°23.950S<br>47°52.367E	AMNH A174651*
	600	Ambohimanana Tolongoina	21°28.557'S<br>47°33.759'E	UADBA 26257, 26377, 26397
	700	Sahamalio	22°26.315'S<br>45°15.648'E	UADBA 26258, 26283
	600	Canyon Singe	22°29.138'S<br>45°23.086'E	UADBA 26259, 26260–26261, 26284, 26401
	650	Ranohira	22°32' S 45°21' E	UADBA 6878
	1300–1400	Amorimbato	23°27.44'S<br>46°20.02'E	UADBA 9772–9776, 9778
	820–840	Ambinany	24°35.6'S<br>46°44.3'E	UADBA 4519
	90	Manantantely	24°59'S<br>46°55.917'E	UMMZ 197651*, UADBA 1409–1415, 1417–1418, 7517
	520	Beampingaratsy Pass	24°28.244'S<br>46°53.525'E	AMNH A181735*
	800–950	Ampasimekieny Pass	24°32'S 46°51'E	UADBA 1422
	500	8 km Northwest Eminiminy/ Andohahela	24°37.55'S<br>46°45.92'E	UADBA 4515, 4517–4518, 4520
Mantidactylus ambreensis	1000	Les Rousettes	12°31'S 49°10'E	UADBA 9056–9057
	650–1150	Atomboka river	12°32.3'S 49°10'S	UMMZ 212426*, 212435*, UADBA 3714
	200	Benavony	13°40'S 48°28'E	UADBA 7222–7224
	450–650	Mandrizavona	13°48.043'S<br>48°44.78'E	AMNH A167482*, UADBA 8397, 8413
	550–750	Besahona	13°54.372'S<br>48°52.425'E	AMNH A167485, UADBA 8394, 8411, 8416
	730–750	Ramena	13°55.071'S<br>48°53.179'E	UADBA 8393, 8396, 8414
	1150	Antsaravy	13°55.560'S<br>48°54.353'E	AMNH A167499–167500, 167565*, UADBA 8408, 8410
	700	Antsahatelo	13°51.588'S<br>48°51.979'E	AMNH A167486, UADBA 8401

	760	Nirhy's cascade Camp	13°51.023'S 48°47.902'E	UADBA 8395, 8398, 8412, 8415
	550	Betaindambo	13°51.932'S 48°49.189'E	UADBA 8399
	350	Analalava	14°35'S 47°42'E	AMNH A50521
	900–1000	Irony	14°44.937'S 48°29.449'E	AMNH A167501*–167504, UADBA 5647–50, 26120, 26222–26224, 26271–26272
	330	Ambohibola	16°38.358'S 47°26.165'E	AMNH A174618*
Mantidactylus ambony	1000	Les Roussetes	12°31'S 49°10'E	UADBA 7225–7226, UADBA 9058
	730–750	Ramena	13°55.071'S 48°53.179'E.	AMNH A167490–167495, 167497, UADBA 8406
	700	Antsahatelo	13°51.588' S 48°51.979' E.	AMNH A167487
	1150	Antsaravy	13°55.560'S 48°54.353'E.	AMNH A167498
	350 m	34 km South from Maevatanana	17°09.092'S 46°51.365'E.	UADBA 5726
Mantidactylus mocquardi	400–670	Salafaina	13°25.405'S 49°41.617'E	UADBA 19916
	350–530	Bezavona	13°31.962'S 49°51.954'E	AMNH A167583, 167585, 167587*, 167584, 167586, UADBA 19597, 19600◎02, 19650, 19652–19653
	970–1300	Sorata	13°41.147'S 49°26.511'E	AMNH 167589, 167597, UADBA19603–19605, 19640
	620–650	Ankitsika	13°52.343'S 49°47.045'E	AMNH A167588, 174622*, UADBA 19654–19655
	250	Ambalafary	14°04'S 48°17'E	UMMZ 21235
	700–1300	Andampimbazaha cascade or Andasimbazaha Marojejy	14°26.05'S 49°45.7'E	AMNH 157114, 157116–157120, UADBA 7774, 7777
	390	Ampanasantongotra Marojejy	14°26.2'S 49°46.5'E	UADBA 7765–7767, 7769, 7772–7773
	650–700	Manantenina river Marojezy	14°26'S 49°46'E	UMMZ 212824*, UADBA 3717, 3719
	1300	Ampanasana Ankolony Marojejy	14°26.2'S 49°46.5'E	AMNH A157111, 157113

	810–980	Ambolokopatrika Betaolana	14°32.302'S 49°26.243'E	AMNH A174621*, PBZT 6641, 6659, 6836, 7042, PBZT/FN6639, UADBA 19596, 19606, 19609–19610, 19646–19647*, 26304–26305
	600–900	Andranomavohely	14°34.165'S 49°16.568'E	UADBA 19648–19649, 26234, 26302–26303
	1260–1550	Anjanaharibe–Sud	14°44.7'S 49°27.7'E	UADBA 8118–8119, 8121, 8123–8124
	785	Tsararano	14°54.667'S 49°41.383'E	PBZT FN6053–6054, 6057, 6110, 6212
	525	Ilampy	15°23.5'S 50°02.783'E	PBZT FAZC10107–10108
	620	Ambatolaidama	15°18.33'S 50°01.24'E	PBZT FN7761
	650	Ambohitsitondroina	15°26'S 49°57.34'E	PBZT RJS342–314
	100	Ankavanana River	15°18.5'S 50°14'E	UMMZ 212881*
	50	Ambodiriana	16°40.469'S 49°42.167'E	AMNH A174652*
	600	Ambatovaky	16°46.913'S 49°14.438'E	UADBA 17861, 19685
	1150	Rangovalo Zahamena	17°40.5'S 48°45.5'E	UADBA 26235. 26240–26241, 26298
	850	Volontsagana Zahamena	17°42'S 48°46'E	UADBA 26225. 26228–26229, 26242
	340–460	Betampona Reserve	17°54.858'S 49°12.474'E	AMNH A174628*, UADBA 26232–26233, 26236, 26238–26239, 26273–26275. 26278–26279, 26285–26294, 26296–26297
	450	Sandranantitra	18°2.9'S 49°5.5'E	UADBA 12307, 12310–12315
	650–840	Mangerivola	18°11.742'S 48°55.095'E	UADBA 17862, 19684, 19686–19690. 26300
	950	Didy	18°11.858'S 48°34.728'E	UADBA 10112–10119
	900	Iofa	18°41'S 28°26'E	UADBA 10070, 11889
	980	Analamay Ambatovy	18°49.715'S 48°19.960'E	UADBA 9253
	895–950	Mantadia Analamazaotra	18°56'S 48°24'E	UADBA 7312–14, UADBA 11891–11892

	1025	Ampanatovana Lakato	19°02.637'S 48°20.912'E	UADBA 26111
	900	Ankeniheny	19°06'S 48°19.85'E	UADBA 6282–6283
	870–1050	Andranomanaponga	18°49.750'S 48°25.9'E	UADBA 19680–19681
Mantidactylus zolitschka	840	An'ala	18°56'S 48°28'E	UADBA 6965–6966, ZMK 60110, 60112–60116, ZSM 184/2003, 939/2000
Mantidactylus catalai	720	Iatara Andringitra	22°13.333'S 47°01.483'E	UMMZ 212890*
	810–1240	Sahavatoy/Volontsagana	22°13.667'S 47°00.217'E	UADBA 26403–26405
	900	Southwest Ranomafana (Ifanadiana road)	–	AMNH 7881–7882 A133689–133690, 18019 A168364
	1150–1300	Amorimbato	23°27.44'S 46°20.02'E	UADBA 9772–9774; 9782
	800–950	Ampasimekieny Pass	24°32'S 46°51'E	UADBA 1419–1421, 1423
	520	Beampingaratsy Pass	24°28.244'S 46°53.525'E	AMNH A181732*, A181821*
	990–1100	Eminiminy	24°35.04'S 46°44.08'E	UADBA 4513–4514; 4516, 4521, 4523
	820	Ambinany	24°35.6'S 46°44.3'E	UADBA 4522
	125	Manatantely forest	24°39'S 46°55.083'E	UADBA 3706
	850	Manangotry	24°45'S 46°52'E	UMMZ 197676
	20	Sainte Luce	24°45'S 47°11'E	UMMZ 191515–19116
	–	Isaka–Ivondro (next to Andohahela)	–	MNHN 1935.153
	75–300	Nahampoana	24°58'S 46°58'E	UMMZ 197662–19764
Mantidactylus poissoni	600	Ambatovaky	16°46.910'S 49°14.417'E	UADBA 19786
	1150	Rangovalo ridge	17°40.5'S 48°45.5'E	UADBA 26409

	895	Sahaberiana	18°47.503'S 48°25.572'E	UADBA 11899
	1450	Mandraka	18°54.727'S 47°55.174'E	AMNH A174653*, MNHN 1937.1. UADBA 39000
	950	Andasibe	18°57'S 48°26'S	UADBA 7125
	900	Ankeniheny	19°05.850'S 48°19.850'E	UADBA 6876
	950	Manasamena	19°02.637'S 48°20.910'E	UADBA 26411–26412, AMNH A174649–174650
***Mantidactylus danieli* nsp**	1150	Atomboka river	12°32.3'S 49°10'S	UMMZ 212827*, UADBA 3710–3713
	350–400	Salafaina	13°26.257'S 49°43.01'E	AMNH A167590*, AMNH A167591–92, UADBA 19595, 26371
	800	Antsahatelo	13°51.588'S 48°51.979'E	AMNH A167580
	750	Besahona	13°54.372'S 48°52.425'E	AMNH A167177
	750	Ramena river	13°55.071'S 48°53.179'E	AMNH A167582, UADBA 8382
	760	Nirhy's cascade Camp Analabe	13°51.023'S 48°47.902'E	AMNH A167578–167579
	850	Andramanalana	14°24.400'S 49°20.217'E	AMNH A181773*
	180–1200	Bekolosy/Antsahabe River	14°02.5'S 48°18'E	UADBA 3716, 12140, UMMZ 212836*
	250	Ambalafary	14°04'S 48°17'E	UMMZ 212835
	1580	Matsaborimena Trois Lacs	14°19.859'S 48°35.240'E	AMNH A167517, UADBA 26359
	1450	Analapakila	14°26.233'S 48°36.696'E	AMNH A167522, UADBA 26361
	1300	Ampanasana Ankolony Marojejy	14°26.2'S 49°46.5'E	AMNH A157111, 157113, UADBA 7765–7767, 7769, 7772–7773
	600	Ampanasantongotra Marojejy	14°26.2'S 49°46.5'E	UADBA 7770
	880–900	Ambolokopatrika	14°32.302'S 49°26.243'E	UADBA 19593, 26113, 26359–60, 26362, 26364–26371, 26373

	850	Andranomavohely	14°34.165'S 49°16.568'E	UADBA 19594
	950	Irony	14°45.140'S 48°29.690'E	AMNH A167523*, UADBA 26372
	400–785	Tsararano	14°54.667'S 49°41.383'E	AMNH A181731*, PBZT FN 6051, 6055
	525	Ilampy	15°23.500'S 50°02.783'E	PBZT FAZC 10053
Mantidactylus macrotympanum **nsp**	180	Antsahabe	13°43.272'S 48°39.304'E	AMNH A167598
	800	Antsahatelo	13°51.588'S 48°51.979'E	AMNH A167589*
	250	Ambalafary	14°04'S 48°17'E	UMMZ 201418, UMMZ 201416*–201417, UMMZ 213447*
Mantidactylus olgai **nsp**	1700	Sorata pic	13°41.147'S 49°26.511'E	AMNH A167596*
	600	Betaindambo	13°51.932'S 48°49.189'E	AMNH A167557
	700	Besahona	13°54.372'S 48°44.785'E	AMNH A167484, 167554–16755, UADBA 8383, 8388,
	700	Maroamalona	13°51.023'S 48°47.902'E	AMNH A167560, AMNH A167561–167562
	600–730	Ramena	13°55.71'S 48°53.179'E	AMNH A167556, 167558, 167564; UADBA 8386, 8391, 8409, 12771
	1150–1180	Antsaravy	13°55.560'S 48°54.353'E	AMNH 167565*–167576, UADBA 8387, 8389–8390, UADBA 8231, 8375, 8381, 8383–8834, 8387, 8392, 8402–8404, 8407, 8409, 8477, 12773
	760	Nirhy's cascade Camp Analabe	13°51.023'S 48°47.902'E	AMNH A167563
	850	Andramanalana	14°24.400'S 49°20.217'E	AMNH A181726*
	180	Antsahabe	13°43.272'S 48°39.304'E	AMNH A167598
	800	Antsahatelo	13°51.588'S 48°51.979'E	AMNH A167589*

***Mantidactylus tavaratra* nsp**	530	Bezavona	13°31.962'S 49°51.954'E	UADBA 19650
	1240– 1350	Sorata 1	13°41.147'S 49°26.511'E	UADBA 19628, 19630, 19634
	1700	Sorata pic	13°41.147'S 49°26.511'E	AMNH A167593*, 167595–167596, UADBA19618–19625, 19628–19629, 19631–19632, 19634–19639, 19642, 19644, 19656
	980	Sorata 2	13°41.986'S 49°26.687'E	AMNH A 167505*, 167597, UADBA 19617, 19643
	350–670	Salafaina montane	13°26.257'S 49°43.01'E	UADBA 19612–19615
	1200	Bekolosy	14°02'S 48°18'E	UMMZ 212440*, UADBA12154–12158
	1000	Manarikoba Manongarivo	14°02.700'S 48°47.007'E	PBZT FAZC11068, 11112
	1050	Antsahamanitsy Tsaratanana	14°02.700'S 48°55.008'E	PBZT RJS102, 119; 122
	1200	Ambinanitelo Tsaratanana	14°04.807'S 48°59.123'E	UADBA 26356
	2650	Trail to summit of Tsaratanana	14°04.807'S 48°59.123'E	UMMZ 212833
	1950–2000	Matsaborimaiky Tsaratanana	14°09.175'S 48°57.431'E	AMNH A167506*–16709, UADBA 26306, 26109, 26116, 26306, 26312, 26328, 26336
	1300–1550	Andampimbazaha or Andasimbazaha Marojejy	14°26.05'S 49°45.7'E	AMNH A187088*–187089*, A157115, UADBA 3724, 3840, UADBA RAX 11532*–11533*
	1050–1300	Antranohafa Marojejy	14°26.2'S 49°44.5'E	AMNH A157121–15722
	1500–1600	Andranomifototra River Marojejy	14°26.4'S 49°44.5'E	AMNH A157123–157129, UADBA 15771–15772, 15774–15778
	600–930	Andranomavohely	14°34.165'S 49°16.568'E	UADBA 19608, UADBA 26230–26231
	530	Ambodivoara	14°31.873'S 49°25.623'E	UADBA 19682
	1550	Anjanaharibe–Sud	14°44.5'S 49°26.5'E	UADBA 8117
