# Supplementary material for: Revision of the Subgenus Ochthomantis Frogs from Madagascar (Amphibia: Mantellidae) with the Description of Four Species and Resurrection of Mantidactylus catalai and M. poissoni
Source: Animals (Basel). 2023 Sep 3;13(17):2800. doi: 10.3390/ani13172800 (PMC10563080; doi:10.3390/ani13172800)
Supplement: Supplementary file 1 [file animals-13-02800-s001.zip › Table S2-12_measurements.pdf]

Table S2. Measurement data for *Mantidactylus femoralis*. All measurements in mm. BMNH: specimen data from Glaw and Vences (2004). F: female, M: male, SA: subadulte, J: juvenile

Specimen	Sexes	SVL	TD	ED	EN	EST	NS	NN	HW	HL	HDL	RC	FE	TI	FT	TA	EO	EM	EHEAD	T1	TO3	TO5
AMNH A167521[b]	SA	43.3	4.9	4.0	2.0	1.3	1.7	4.4	14.5	19.6	13.4	7.9	21.8	22.5	23.5	10.5	4.0	2.3	5.7	1.5	5.7	6.1
AMNH A167580[b]	J	23.8	-	-	-	-	-	-	-	-	-	-	-	-	-	-	-	-	-	-	-	-
BMNH 1947.2.22.65[a]	F	51.2	3.7	5.6	4.2	◎	2.4	4.3	16.4	17.8	13.5	-	-	-	-	-	-	-	-	-	-	-
AMNH A167582	F	51.4	6.2	9.2	4.2	0.9	1.7	4.5	18.7	21.7	14.2	10.0	27.2	27.6	28.0	13.3	9.2	3.4	8.4	1.8	7.3	7.7
AMNH A174623[b]	F	45.4	3.7	5.2	3.8	2.1	2.4	4.1	14.3	19.8	13.6	8.7	23.2	23.8	25.4	11.7	6.8	2.6	6.7	1.6	6.4	7.6
AMNH A174646[b]	F	47.9	3.5	5.3	4.3	2.2	2.1	3.9	16.3	20.1	15.3	9.8	27.6	29.3	29.3	13.5	7.1	2.5	7.1	1.8	7.6	8.7
UADBA 1410	F	45.1	4.4	6.1	4.4	1.8	1.6	4.9	17.3	22.0	15.1	8.6	26.0	28.7	28.0	13.2	9.3	3.5	8.0	1.7	6.7	7.9
UADBA 1411	F	50.7	4.0	6.7	5.3	2.2	1.6	4.8	18.0	22.4	14.8	10.0	25.7	29.3	27.6	13.4	9.1	2.8	8.4	2.1	6.8	8.8
UADBA 1415	F	43.4	4.1	6.4	4.6	2.1	1.6	4.9	17.3	21.5	14.1	9.5	25.9	28.6	26.0	11.0	8.3	3.3	7.6	2.1	7.2	8.2
UADBA 1417	F	51.8	4.0	6.7	4.3	2.5	3.0	5.5	17.8	23.2	13.8	9.1	25.5	28.0	26.2	12.0	9.5	3.5	8.0	1.9	7.2	8.0
UADBA 1418	F	54.1	4.3	6.6	4.8	2.0	2.6	5.2	17.9	23.0	15.1	9.4	28.0	29.2	26.7	12.7	9.3	2.7	8.2	2.2	7.5	8.6
UADBA 1422	F	59.0	4.5	7.4	5.0	3.1	2.6	5.4	19.6	23.9	16.9	10.9	29.8	30.1	30.0	13.1	9.9	3.3	8.1	2.1	8.0	9.0
UADBA 3710	F	49.0	4.2	6.3	4.0	1.6	2.1	5.0	17.4	19.6	14.0	9.3	26.7	26.5	25.0	12.8	8.6	3.5	9.0	1.8	6.8	7.0
UADBA 4515	F	47.2	4.0	5.9	4.6	2.5	3.0	4.1	17.0	22.0	13.8	8.6	24.5	26.0	26.0	12.1	8.8	2.6	7.1	1.7	5.9	7.2
UADBA 5828	F	45.7	4.0	6.1	4.5	2.1	3.0	5.0	10.9	21.1	14.1	8.4	24.0	25.2	25.5	11.4	7.7	2.9	7.0	1.4	5.6	6.8
UADBA 6280	F	51.6	4.2	7.1	4.2	2.0	3.0	5.0	17.9	21.7	15.9	10.2	28.7	28.8	30.0	14.0	9.2	3.0	7.8	2.1	7.7	8.6
UADBA 6281	F	55.8	3.0	6.1	4.4	2.0	3.2	5.3	18.1	22.4	16.6	10.2	30.0	30.1	30.4	13.9	9.2	2.9	7.4	2.2	7.8	9.8
UADBA 7516	F	50.1	3.6	5.8	4.0	2.2	2.9	4.7	17.1	21.4	15.1	10.4	27.6	29.5	29.7	12.1	7.4	2.8	7.4	2.1	7.3	8.9
UADBA 7517	F	54.5	4.5	6.7	4.7	2.4	2.4	5.1	17.9	23.2	14.6	8.4	24.4	27.2	27.3	13.3	9.3	3.2	8.2	2.2	6.6	7.9
UADBA 7778	F	45.2	3.5	5.8	3.5	2.3	2.4	4.0	15.1	24.1	13.5	8.9	25.0	25.5	26.5	11.5	7.6	2.4	7.0	1.4	6.3	7.5
UADBA 8527	F	48.9	3.8	5.8	4.0	2.2	2.3	4.1	14.7	20.0	14.0	9.2	25.9	26.3	27.6	11.0	7.5	2.6	6.8	1.7	7.3	8.3
UADBA 9251	F	54.0	4.4	6.6	4.4	2.3	2.5	5.3	17.4	22.6	16.1	10.8	25.8	27.7	28.5	13.0	8.6	2.8	7.6	2.1	7.5	9.6
UADBA 9775	F	46.5	3.8	5.9	4.0	2.3	2.8	4.2	15.9	20.6	14.0	8.0	24.3	24.5	25.0	12.6	8.1	2.6	6.9	1.8	6.4	7.7
UADBA 9777	F	43.3	3.2	4.6	3.3	1.5	2.0	3.7	14.7	17.3	13.1	7.8	20.6	21.6	23.4	9.8	7.1	2.6	5.9	1.4	6.1	7.1
UADBA 9778	F	41.8	3.5	4.7	3.7	1.7	2.2	3.7	14.1	18.6	12.6	7.9	21.6	22.4	23.6	10.6	6.1	2.2	5.5	1.6	6.2	7.2
UADBA 9784	F	41.1	3.5	5.1	3.6	2.2	2.6	4.2	13.7	18.0	11.7	8.1	20.6	21.6	23.7	12.5	7.3	2.6	5.3	1.8	5.9	6.0
UADBA 9787	F	37.4	2.8	4.5	3.1	1.3	1.1	3.5	12.7	16.4	11.7	6.5	20.0	21.0	22.2	11.0	6.0	2.3	7.2	1.6	6.1	6.2
UADBA 11886	F	39.1	3.3	4.8	3.4	2.0	2.3	3.8	13.2	17.8	11.5	8.3	21.2	23.4	22.0	12.0	6.9	2.2	5.8	1.7	5.4	6.3

UADBA 11977	F	57.4	4.1	6.6	5.0	1.9	2.7	4.9	18.3	24.3	15.8	9.8	27.3	30.6	30.6	13.8	9.4	3.3	8.1	1.9	9.1	10.0
UADBA 12306	F	51.2	4.2	6.7	4.6	2.2	2.6	4.9	18.0	22.4	15.4	10.3	30.4	32.7	30.7	13.6	9.0	3.3	8.0	2.3	8.0	8.7
UADBA 12453	F	47.4	3.7	5.3	4.3	2.4	2.4	4.9	16.4	20.6	15.8	9.0	26.5	26.7	28.2	13.0	8.0	3.1	7.0	1.4	7.9	8.5
UADBA 19657	F	55.5	4.4	7.1	7.0	2.4	3.3	4.8	18.0	23.0	16.0	10.1	28.7	29.8	30.7	13.2	9.1	2.6	8.4	2.6	7.6	9.0
UADBA 19663	F	44.1	3.5	5.4	4.0	2.0	2.4	4.4	14.6	18.4	14.4	8.7	23.6	25.8	25.8	11.5	7.6	2.6	6.9	1.6	7.0	7.7
UADBA 19665	F	55.5	4.2	7.1	4.4	2.7	3.0	4.8	18.0	22.3	15.2	10.1	27.8	30.3	28.6	12.0	9.4	3.4	8.2	2.3	7.5	9.3
UADBA 19666	F	53.4	3.7	7.0	4.4	2.9	2.9	4.2	17.6	21.7	15.8	10.6	26.6	29.4	30.1	12.4	8.7	3.2	7.8	2.0	7.9	8.4
UADBA 19667	F	59.4	4.3	7.6	5.1	2.7	2.9	4.7	19.7	25.7	16.3	10.0	29.8	30.5	30.7	13.6	9.8	3.1	9.0	2.4	8.2	9.3
UADBA 19668	F	52.6	4.3	6.6	4.9	1.8	2.7	4.3	17.9	21.6	15.4	10.2	26.6	28.2	29.6	12.5	9.4	3.3	7.6	2.1	7.2	8.6
UADBA 19669	F	58.0	4.8	7.1	5.3	2.0	2.8	4.8	19.4	22.8	15.5	11.1	28.4	30.4	29.2	12.8	9.8	3.1	8.6	2.3	7.3	8.8
UADBA 19670	F	62.4	4.7	7.7	5.4	2.1	2.2	4.8	20.3	25.0	16.3	12.3	28.4	30.3	30.7	13.0	10.3	3.4	9.0	2.4	8.2	9.5
UADBA 19675	F	57.4	4.6	7.2	4.6	2.5	1.8	5.0	19.5	24.2	17.2	10.8	27.6	32.3	32.1	15.5	9.4	3.5	9.4	2.5	8.4	9.6
UADBA 19676	F	53.7	3.8	6.4	5.2	2.8	2.8	4.9	18.5	23.8	15.5	10.9	28.8	30.3	29.2	13.3	9.4	3.2	8.3	2.2	7.7	9.3
UADBA 19677	F	41.8	3.7	5.4	3.8	1.8	2.7	3.9	14.3	18.0	14.0	8.3	22.8	24.6	24.4	11.0	7.3	2.5	6.3	1.6	6.1	7.3
UADBA 19691	F	52.4	4.3	6.2	4.9	2.6	2.6	5.0	19.3	23.0	15.8	9.9	29.8	31.6	30.8	13.4	9.2	3.2	7.7	2.0	7.6	9.2
UADBA 19693	F	45.6	3.8	5.7	4.6	2.1	2.6	4.3	16.0	20.2	14.8	9.5	27.3	27.8	28.1	11.6	7.5	2.8	7.2	1.5	7.0	8.1
UADBA 19694	F	49.7	3.6	6.2	4.5	2.0	1.4	5.0	17.1	22.1	15.4	9.4	27.5	30.6	29.2	14.4	9.0	3.0	7.3	1.7	8.0	8.7
UADBA 19696	F	47.9	3.2	5.3	4.0	2.4	2.4	4.3	14.7	20.0	13.3	7.1	21.9	24.4	24.3	11.0	8.0	2.6	6.7	1.1	6.6	7.5
UADBA 20479	F	51.0	4.0	6.1	2.9	1.5	2.3	3.0	17.3	21.4	14.4	8.0	26.5	27.3	27.4	13.0	8.4	2.6	7.7	1.9	7.0	8.2
UADBA 26380	F	43.0	3.6	5.6	3.4	2.0	2.3	3.9	14.3	18.1	12.6	8.3	22.7	24.4	24.0	11.1	7.2	2.3	5.8	1.4	6.1	7.6
UADBA 26281	F	45.2	3.8	5.9	3.7	2.5	2.0	4.0	15.1	20.4	13.4	8.0	20.8	23.0	24.4	9.9	7.1	2.9	6.6	1.6	5.6	6.8
UADBA 26282	F	55.9	4.1	6.4	4.8	2.8	2.4	5.0	18.8	23.6	16.4	9.4	26.7	26.8	28.7	12.9	9.4	3.2	8.1	2.0	7.3	8.0
UADBA 26283	F	46.2	3.8	5.8	3.8	2.6	3.0	4.4	16.5	19.6	13.4	8.4	23.3	24.2	25.1	11.5	7.6	3.1	7.0	1.8	6.3	7.6
UADBA 26284	F	45.7	3.8	5.8	4.4	2.5	3.1	4.4	15.1	19.5	13.0	7.3	21.5	22.5	23.7	10.9	7.0	2.3	6.6	1.4	6.0	7.0
UADBA 26266	F	40.1	2.8	4.4	3.2	2.0	1.7	3.5	13.4	16.2	12.1	8.1	23.0	24.7	23.1	9.5	6.4	2.2	5.9	1.4	6.3	6.6
UADBA 26363	F	52.6	4.2	6.6	4.5	2.2	3.3	4.9	18.1	22.5	16.1	9.6	26.9	28.3	28.6	13.6	9.4	3.2	8.3	1.8	7.5	8.5
UADBA 26378	F	47.3	4.0	6.4	4.3	2.5	2.3	4.3	15.8	20.5	14.5	9.1	25.6	26.5	26.7	12.0	7.6	2.6	7.3	1.6	7.1	8.1
UADBA 26379	F	54.3	4.1	6.7	4.7	2.8	2.6	5.4	18.9	24.6	16.0	9.3	28.9	29.6	30.1	15.3	10.0	3.6	9.5	2.0	9.2	9.8
UADBA 26385	F	51.4	4.3	6.2	4.0	2.5	2.1	4.0	16.9	20.7	15.4	9.8	25.8	29.3	29.2	12.8	8.5	2.8	7.9	2.6	7.3	8.5
UADBA 26386	F	52.3	3.5	6.5	4.3	2.1	2.7	4.4	17.3	21.6	14.4	8.2	26.6	29.6	28.4	12.8	9.1	2.9	7.6	2.0	7.7	7.8
UADBA 26387	F	52.5	3.4	6.3	4.9	2.1	2.8	4.6	18.3	24.1	15.4	8.6	26.2	29.2	27.8	13.3	9.0	3.2	8.7	2.0	7.1	7.5
UADBA 26388	F	45.2	3.6	5.7	4.1	2.4	2.8	4.2	15.8	18.0	14.4	9.2	25.3	27.4	27.6	11.6	8.1	2.8	7.3	1.6	7.2	8.6
UADBA 26389	F	44.2	3.4	5.6	3.4	2.7	2.7	3.6	13.6	18.8	14.5	8.7	23.4	24.7	26.8	11.8	7.1	2.4	6.7	1.8	6.8	8.0

UADBA 26390	F	50.6	4.1	6.2	4.3	2.5	2.5	4.8	16.6	22.7	14.6	10.6	27.8	28.1	28.1	13.0	8.7	3.0	8.2	2.2	7.2	8.5
UADBA 26391	F	54.0	4.6	6.0	4.5	1.6	2.8	4.8	18.7	22.8	15.1	9.2	28.3	28.8	28.9	13.6	9.5	3.6	8.6	1.8	7.5	7.9
UADBA 26392	F	55.3	4.2	7.0	4.6	2.7	2.9	4.9	17.2	22.7	16.0	10.0	29.2	30.5	29.6	14.3	8.6	3.2	8.3	2.0	7.7	7.8
UADBA 26395	F	52.8	4.0	7.2	4.4	2.1	1.5	4.0	18.2	22.0	13.7	9.8	25.2	27.8	27.4	13.0	9.6	2.7	8.1	2.1	6.6	7.6
UADBA 26396	F	51.4	4.3	6.4	4.4	1.8	2.1	4.2	17.4	20.5	14.8	9.3	26.2	28.3	27.2	12.9	8.4	2.8	7.1	2.1	7.7	8.7
UADBA 26397	F	46.6	3.5	5.3	4.1	1.9	1.0	4.3	16.0	20.4	14.2	8.4	26.4	26.4	25.4	11.6	8.3	2.8	7.0	1.6	6.7	8.2
AMNH A167581[b]	M	40.2	4.7	7.5	3.4	1.4	1.8	3.0	13.6	17.8	12.2	7.1	19.3	20.5	21.6	9.7	7.5	2.4	6.3	2.2	5.8	6.1
AMNH A174627	M	41.0	4.1	4.9	3.1	1.7	2.0	3.8	12.6	18.6	11.8	7.9	19.2	20.7	22.1	10.0	5.6	2.1	6.6	1.6	5.9	6.3
UADBA 1409	M	39.4	3.4	4.3	3.8	1.3	1.6	4.1	14.4	17.3	12.1	8.7	21.4	23.0	23.5	11.4	6.9	2.3	6.5	1.3	6.6	7.0
UADBA 1412	M	37.0	3.6	4.4	3.6	1.6	2.1	4.3	13.0	16.7	12.1	6.9	20.2	21.0	20.5	10.2	6.3	2.3	7.4	1.6	5.5	7.0
UADBA 1413	M	36.6	3.1	4.2	3.3	1.4	1.8	3.7	13.3	16.8	10.8	6.6	19.4	21.5	20.8	9.0	6.8	2.4	6.4	1.3	5.4	6.6
UADBA 1414	M	40.2	4.8	5.0	3.6	2.3	1.5	4.0	14.3	17.7	11.6	8.6	20.6	22.0	21.0	10.2	7.3	3.2	8.4	1.6	6.1	6.5
UADBA 3720	M	36.0	4.0	4.7	3.5	1.6	2.0	4.0	11.4	16.0	11.3	6.6	18.9	18.5	18.8	8.1	5.7	2.0	6.0	1.2	5.2	5.9
UADBA 4518	M	36.9	4.5	5.6	3.1	2.3	2.5	3.6	12.7	16.7	11.6	7.3	20.4	20.3	20.5	10.4	6.2	2.6	7.3	1.4	5.4	6.0
UADBA 5728	M	35.3	4.0	4.4	2.9	1.3	2.2	3.4	12.6	15.6	11.0	6.4	17.7	18.0	17.6	8.9	4.6	2.7	6.6	1.5	4.7	5.3
UADBA 7241	M	39.0	4.3	5.3	3.5	1.9	2.4	4.3	13.6	18.0	12.4	6.8	21.3	22.0	21.0	11.2	6.3	2.0	5.6	1.6	5.7	6.4
UADBA 7278	M	38.4	4.2	5.3	3.0	1.6	2.3	4.0	11.8	18.0	11.6	6.7	19.3	20.4	21.3	9.5	6.6	2.2	6.2	1.4	5.8	6.2
UADBA 7515	M	40.0	4.4	5.2	3.8	1.8	2.2	4.4	14.3	19.1	12.0	8.0	20.0	21.2	22.3	10.0	6.6	2.4	5.8	1.7	5.7	6.4
UADBA 7768	M	39.0	4.4	5.6	3.3	0.4	1.6	3.8	13.6	18.2	11.8	6.6	19.5	21.1	21.2	9.6	6.2	2.3	6.5	1.4	5.6	6.6
UADBA 8525	M	36.6	3.9	4.5	3.1	1.7	2.3	3.6	12.0	17.1	10.8	6.8	19.0	20.0	21.0	10.5	6.2	2.0	5.4	1.0	5.7	6.4
UADBA 9252	M	38.6	4.5	5.4	3.5	1.8	2.3	4.2	13.4	17.5	12.3	7.1	20.3	21.3	21.3	8.2	7.3	2.1	6.2	1.7	5.6	6.1
UADBA 9254	M	41.6	4.2	4.8	3.5	2.1	2.2	4.2	13.2	18.0	12.8	7.5	20.0	21.3	21.7	10.0	6.5	2.2	6.8	1.8	5.2	6.1
UADBA 9776	M	35.4	4.1	5.4	2.7	1.6	2.2	4.0	11.7	16.6	11.0	6.1	16.6	17.6	18.4	8.5	6.1	1.6	6.1	1.5	5.1	5.4
UADBA 9781	M	36.5	3.4	3.8	3.3	2.0	1.8	3.5	12.1	15.5	11.7	6.7	18.2	18.7	19.6	8.3	5.1	2.3	6.4	1.4	5.0	6.0
UADBA 9788	M	33.8	3.8	4.8	3.0	1.1	1.5	3.4	11.4	14.1	10.8	5.7	17.3	17.8	19.3	8.6	5.5	2.2	4.5	1.0	5.3	5.8
UADBA 11887	M	33.0	3.0	4.2	2.5	1.3	1.8	3.7	10.7	14.0	10.0	5.7	16.8	18.1	18.5	8.7	5.4	1.7	4.3	1.2	5.6	6.1
UADBA 11890	M	41.1	5.1	5.5	3.7	1.7	2.4	4.3	14.1	18.1	12.4	7.7	20.6	21.2	22.0	10.0	7.0	2.2	6.5	1.5	5.6	6.3
UADBA 11975	M	37.8	3.2	4.5	3.7	1.7	1.9	3.0	12.7	17.3	11.3	7.2	19.7	20.4	21.3	10.6	6.6	2.2	5.8	1.7	5.4	6.6
UADBA 11976	M	37.4	3.3	4.1	2.8	2.0	2.2	2.6	12.7	16.5	11.7	6.9	20.0	20.3	19.8	9.2	5.6	2.0	5.3	1.1	5.6	6.0
UADBA 12308	M	35.3	3.3	3.9	2.7	2.1	1.7	2.8	12.1	16.2	10.8	7.3	19.0	20.0	20.3	10.3	5.7	2.3	5.7	1.5	5.7	6.2
UADBA 12309	M	36.4	3.8	4.5	3.2	2.2	2.2	3.1	13.0	17.8	11.7	7.1	19.7	20.7	20.0	8.8	6.6	2.5	6.3	1.7	4.6	5.8
UADBA 12451	M	38.7	4.3	5.2	3.8	1.6	2.0	3.7	13.0	18.0	12.4	6.8	18.6	19.5	21.5	9.0	6.2	1.7	4.9	1.0	5.3	6.4
UADBA 19658	M	39.8	4.5	5.8	4.0	1.9	2.8	4.0	14.0	19.1	12.8	7.3	21.6	22.4	22.8	10.1	7.2	2.2	6.4	1.4	5.6	6.6
UADBA 19659	M	38.4	3.8	5.0	3.4	1.5	2.3	3.7	12.5	17.5	11.6	7.5	20.2	21.6	21.0	10.0	6.3	2.0	5.6	1.2	5.9	6.0
UADBA 19660	M	42.4	5.0	5.8	3.9	1.7	2.6	3.9	15.2	19.0	13.4	7.9	20.9	22.0	23.5	10.0	7.0	2.3	6.7	1.6	6.0	6.8
UADBA 19661	M	39.0	4.2	4.8	3.5	1.5	2.3	3.4	12.6	17.7	12.1	8.0	20.3	21.5	22.7	10.1	6.8	2.3	6.2	1.4	5.7	6.3

UADBA 19662	M	37.5	4.2	4.8	3.4	1.8	2.4	3.6	12.5	18.0	11.4	7.5	19.8	20.8	21.2	9.5	6.0	1.8	5.4	1.5	5.6	6.3
UADBA 19671	M	35.8	3.4	4.6	3.2	1.9	1.8	3.3	11.0	16.0	11.0	6.4	19.0	19.8	21.2	9.0	5.6	2.0	5.6	1.4	5.0	5.8
UADBA19672	M	38.8	4.2	5.1	3.6	1.5	2.2	3.8	13.7	18.4	11.6	7.4	20.5	21.6	21.4	10.4	6.9	2.5	6.6	1.4	5.4	6.0
UADBA 19673	M	38.9	4.2	5.9	3.4	1.0	2.6	3.6	13.1	18.4	12.3	7.8	20.7	21.8	22.6	10.4	6.5	2.3	6.2	1.4	6.2	6.9
UADBA 19674	M	39.7	4.7	5.8	3.6	1.7	1.0	3.6	14.2	18.1	11.7	7.6	19.2	21.3	21.4	9.3	6.8	2.3	7.0	1.8	6.0	6.6
UADBA19679	M	43.4	4.1	5.6	3.6	1.9	2.1	3.7	13.3	19.3	12.7	6.6	20.0	20.7	22.2	11.0	6.8	2.2	6.2	1.3	5.6	6.4
UADBA 19886	M	34.6	3.3	4.4	3.4	1.4	2.0	3.5	11.9	15.5	11.3	6.4	17.4	18.7	19.1	11.0	5.6	1.7	4.9	1.0	5.4	6.4
UADBA 20478	M	37.8	3.6	4.1	2.9	1.4	2.1	3.0	11.8	16.6	11.2	7.4	19.1	19.8	20.8	10.3	6.1	2.0	5.6	1.4	5.3	6.6
UADBA 26249	M	38.3	4.1	4.4	3.0	1.6	2.2	3.0	11.6	16.6	11.9	6.5	18.7	19.8	22.4	9.4	6.2	2.2	5.8	1.3	5.7	6.5
UADBA 26250	M	38.6	3.8	4.1	2.9	1.2	1.0	3.5	12.2	16.9	12.2	7.5	20.0	21.7	21.8	9.3	6.5	2.0	6.2	1.2	5.0	5.8
UADBA 26251	M	39.4	4.0	5.0	4.0	1.6	2.7	3.1	13.5	17.9	12.8	7.4	18.6	22.1	23.0	10.4	6.6	2.4	7.0	2.1	5.6	6.9
UADBA 26252	M	36.8	4.3	5.2	3.4	1.8	2.2	4.0	12.3	16.7	11.3	6.7	17.2	19.4	20.6	9.0	6.4	2.0	6.3	1.5	5.2	5.8
UADBA 26255	M	36.0	3.8	4.4	2.8	1.6	2.6	4.2	11.9	15.3	10.2	6.2	16.6	17.8	18.2	8.4	6.2	2.0	5.2	1.3	5.0	5.6
UADBA 26257	M	33.6	3.6	4.0	3.3	1.7	1.8	3.8	11.8	15.8	11.0	6.3	18.5	19.5	18.3	7.2	5.8	1.9	6.2	1.4	4.5	5.5
UADBA 26258	M	38.6	4.8	4.8	3.3	2.5	2.2	3.4	12.5	17.7	11.4	7.2	18.7	18.6	20.0	9.6	6.4	2.3	8.0	1.9	4.9	6.2
UADBA 26259	M	36.0	4.0	5.0	3.2	1.6	2.4	3.8	12.6	16.6	10.0	5.9	15.8	17.6	19.0	8.2	6.8	2.0	5.4	1.2	5.2	5.4
UADBA 26260	M	34.9	3.5	5.1	2.8	1.8	2.3	4.1	12.1	15.0	10.8	6.8	17.3	18.1	18.7	9.9	5.5	2.2	8.0	1.4	4.7	6.2
UADBA 26261	M	35.8	3.8	4.3	3.0	1.8	2.2	3.0	12.0	17.2	10.1	5.6	17.3	17.0	17.7	8.4	5.6	2.1	6.3	1.1	4.8	5.3
UADBA 26262	M	36.5	4.3	4.5	3.3	1.8	2.4	3.2	12.5	17.0	12.0	5.9	18.2	18.5	21.3	8.9	5.9	2.0	5.7	1.4	5.7	6.1
UADBA 26263	M	39.6	4.1	5.0	3.0	2.0	2.0	3.0	13.2	18.4	11.6	6.6	20.1	20.2	21.1	9.2	6.0	2.3	6.4	1.5	5.6	6.0
UADBA 26264	M	36.4	3.6	4.7	3.1	1.4	2.6	3.3	12.6	16.5	12.1	7.2	20.1	21.5	21.8	9.7	6.3	2.3	6.2	1.5	5.6	6.1
UADBA 26265	M	39.4	4.3	5.4	3.7	1.9	2.1	3.3	13.4	17.3	11.9	7.5	21.6	21.8	21.7	10.6	6.6	2.2	6.3	1.5	5.6	6.5
UADBA 26267	M	36.5	4.1	5.4	3.5	1.7	2.4	4.1	13.0	17.2	11.3	7.4	19.8	20.8	20.6	11.5	6.6	2.4	6.3	1.4	5.5	6.1
UADBA 26268	M	40.2	4.0	5.4	3.6	1.8	2.4	3.8	13.2	18.7	12.6	6.4	21.7	22.4	22.5	10.1	6.4	2.2	5.8	1.5	6.0	6.7
UADBA 26269	M	36.6	3.6	4.3	3.1	1.9	2.4	3.4	12.6	17.3	12.4	7.2	20.3	20.5	21.2	8.8	6.1	2.2	5.5	1.3	5.6	6.3
UADBA 26270	M	37.5	3.8	4.5	3.1	1.7	2.5	3.8	13.1	17.3	12.9	7.6	21.4	21.4	23.8	10.1	5.8	2.1	5.6	1.3	6.1	6.7
UADBA 26375	M	38.6	4.5	5.3	4.1	2.0	2.4	3.6	13.1	18.2	12.7	6.7	20.2	21.8	23.1	9.8	6.9	2.1	6.1	1.5	5.7	7.0
UADBA 26376	M	37.2	4.0	5.0	3.1	1.6	1.9	3.1	11.6	16.6	10.8	6.0	15.8	18.6	19.3	9.3	5.6	1.8	5.1	1.3	4.8	5.6
UADBA 26402	M	40.4	4.1	5.7	3.1	2.1	2.3	3.3	13.6	17.0	12.2	6.9	21.2	21.8	23.2	8.8	6.8	2.2	6.5	1.7	5.7	6.8

[a]Lectotype

[b] ID Molecular

Table S3. Measurements data for *Mantidactylus ambreensis*. All measurements in mm. F: female, M: male, J: juvenile, Sa: subadult.

Specimen	Sexes	SVL	TD	ED	EN	EST	NS	NN	HW	HL	HDL	RC	FE	TI	FT	TA	EO	EM	EHEAD	T1	TO3	TO5
AMNH A50521	J	12	2.4	3.4	1.7	0.9	1.1	2.7	◎	8.7	27.4	◎	◎	14.5	16.2	◎	7.8	◎	5.4	1.4	4	4.3
UADBA 7223	J	23.7	◎	◎	◎	◎	◎	◎	◎	◎	◎	◎	◎	◎	◎	◎	◎	◎	◎	◎	◎	◎
UADBA 8414	SA	31.4	3.1	4.0	2.9	1.8	1.7	3.0	10.8	12.6	9.6	6.8	15.6	18.8	17.4	7.7	5.5	1.6	4.7	1.2	4.4	5.3
MNHN 1893.241[a]	F	42.2	3.4	4.6	3.3	-	1.5	3.2	12.3	14.4	11.7	◎	◎	◎	20.0	◎	◎	◎	◎	◎	◎	◎
UADBA 5650	F	39.0	3.6	5.3	3.5	1.7	2.6	3.7	13.0	16.7	12.0	7.2	19.3	19.8	19.3	11.1	7.0	2.4	5.7	1.3	4.8	5.4
UADBA 8394	F	40.5	3.7	5.5	3.3	1.9	2.5	3.8	13.8	16.4	12.6	8.0	20.5	23.5	21.3	10.3	7.0	2.1	6.3	1.2	4.7	5.4
UADBA 8396	F	38.4	2.8	5.3	3.0	1.7	2.0	3.4	11.7	15.4	10.4	7.7	19.0	20.6	19.0	9.9	6.2	1.7	5.5	1.6	4.7	5.9
UADBA 8398	F	38.2	2.7	4.5	2.8	1.6	2.7	3.4	12.0	15.0	12.8	8.6	19.3	22.8	20.5	11.5	6.6	1.8	5.5	1.4	4.9	5.8
UADBA 8399	F	39.1	2.7	5.0	3.5	1.4	2.1	3.3	11.6	14.0	13.0	7.0	18.6	20.5	20.3	10.2	6.1	1.9	5.4	1.5	5.0	6.2
UADBA 8401	F	38.3	3.3	5.3	3.3	1.9	2.4	4.0	11.9	15.7	11.6	7.8	19.1	21.2	18.4	10.0	7.1	2.1	6.0	1.8	5.0	6.3
UADBA 8412	F	42.0	3.0	5.4	3.0	1.6	2.0	3.4	13.4	16.4	11.4	7.6	19.4	22.1	20.6	9.8	6.9	2.2	5.9	1.4	4.7	5.4
UADBA 9056	F	39.7	3.6	5.7	3.8	1.5	1.9	3.4	13.2	14.8	10.6	8.2	19.5	20.2	19.3	10.7	7.5	2.6	6.0	1.3	4.9	5.5
UADBA 9057	F	41.6	3.4	5.0	3.6	1.9	2.4	3.7	13.7	16.4	12.2	7.3	20.6	20.9	21.6	9.7	7.5	2.3	7.2	1.3	6.0	6.6
AMNH A167482[b]	M	38.1	5.1	4.4	2.8	1.6	1.9	3.1	12.0	16.6	11.2	6.2	17.9	19.7	19.5	9.2	6.5	2.3	5.6	1.7	5.1	5.8
AMNH A167485	M	35.0	4.4	5.0	2.5	1.9	1.5	2.9	11.0	15.9	10.0	6.3	17.4	18.5	18.0	8.3	6.3	1.9	5.5	1.4	4.4	5.1
AMNH A167486	M	34.7	5.0	4.5	2.8	1.5	1.1	2.8	9.9	15.2	10.0	6.1	17.7	18.7	18.0	8.6	5.8	1.8	5.0	1.1	4.5	5.2
AMNH A167501[b]	M	33.4	4.4	4.2	2.5	1.8	2.2	2.5	10.6	15.6	9.0	6.1	16.2	17.6	16.7	8.7	6.0	1.9	5.3	1.0	4.0	4.9
AMNH A 167504	M	34.6	5.0	5.0	3.0	1.6	1.5	3.2	11.6	16.0	11.7	6.1	17.6	19.0	19.7	8.1	6.2	2.4	5.6	1.5	5.1	6.0
UADBA 3714	M	35.0	4.2	4.2	2.8	1.0	1.8	3.7	11.5	14.8	10.7	7.2	17.6	18.8	19.8	8.4	5.8	1.8	5.6	1.4	5.1	5.8
UADBA 5647	M	35.1	5.0	4.6	3.0	1.5	1.3	3.1	10.8	14.7	10.1	6.7	17.0	19.0	17.2	9.2	6.4	1.7	5.2	1.4	4.1	4.7
UADBA 5648	M	35.6	4.7	4.4	3.1	1.9	2.6	3.4	11.7	15.6	10.7	6.4	17.5	18.0	17.8	8.4	6.9	1.8	5.1	1.4	4.6	5.3
UADBA5649	M	36.0	5.0	5.0	3.7	1.8	2.6	3.5	11.7	15.4	11.0	6.8	16.5	17.6	17.8	9.0	7.0	1.8	5.2	1.4	4.7	5.3
UADBA 7222	M	33.8	4.0	5.0	2.5	1.6	2.3	3.7	10.4	13.8	10.2	6.3	16.2	17.9	16.9	9.0	5.2	1.8	5.2	1.2	4.6	5.4
UADBA7224	M	37.0	4.2	5.3	3.2	1.6	2.0	3.3	11.4	16.0	10.3	6.8	17.9	19.7	18.2	10.5	5.1	2.1	5.5	1.4	4.5	5.4
UADBA 8393	M	39.0	4.8	4.6	3.4	2.0	1.7	3.4	12.4	17.1	12.0	6.8	19.8	20.4	20.1	8.7	7.0	2.3	5.8	1.8	4.9	5.8
UADBA 8395	M	35.2	5.4	5.1	3.0	1.4	1.8	3.3	11.7	16.0	9.6	7.0	17.2	18.5	18.0	9.0	6.7	2.1	5.3	1.2	4.7	5.8
UADBA 8408	M	33.4	5.1	4.4	2.3	1.6	1.8	3.3	10.0	14.8	9.8	5.8	15.4	17.1	16.1	7.9	5.9	1.6	4.8	1.4	4.3	4.8
UADBA 8410	M	33.6	5.2	4.5	2.7	1.6	2.0	3.3	10.7	14.8	10.3	7.1	16.3	18.8	17.2	9.4	5.6	1.8	4.5	1.1	4.4	5.1
UADBA 8411	M	35.8	4.6	4.8	3.1	1.5	1.8	3.4	11.7	15.1	10.3	6.7	17.0	19.2	16.5	10.0	6.6	1.9	5.6	1.8	4.2	5.0
UADBA 8413	M	36.7	4.3	4.7	3.3	1.5	2.0	3.4	11.8	17.1	11.3	6.8	18.6	20.5	19.2	10.6	6.6	2.2	5.5	1.4	5.0	6.0

UADBA 8416	M	35.3	4.1	4.4	3.2	2.1	2.0	3.3	11.0	15.6	11.6	7.9	17.3	19.3	20.0	10.5	6.3	1.9	5.3	1.6	5.1	5.9
UADBA 26222	M	35.3	5.4	5.0	3.1	1.8	2.4	3.8	11.6	16.4	10.3	5.5	16.4	18.1	17.3	8.0	6.3	2.0	4.8	1.3	4.0	4.9
UADBA 26223	M	38.4	5.9	5.3	3.7	1.6	2.6	3.6	12.0	17.4	12.0	6.8	18.5	18.4	19.0	8.1	6.8	1.9	5.4	1.3	4.4	5.6
UADBA 26224	M	34.2	5.1	5.0	4.0	1.6	2.2	3.7	11.4	15.5	11.6	7.3	18.0	19.5	19.3	8.7	6.4	1.7	4.8	1.1	4.4	5.3
UADBA 26399	M	35.1	3.4	4.4	3.2	1.3	2.0	3.7	11.9	15.6	10.8	6.9	16.6	21.3	20.2	9.5	5.7	2.2	6.4	1.4	5.2	6.3
UADBA 26400	M	33.3	3.3	4.3	2.8	1.1	1.6	3.6	11.6	14.5	11.7	7.0	18.6	18.7	19.4	8.3	5.2	1.8	5.5	1.2	5.3	5.8
UADBA 26401	M	36.7	3.6	4.6	3.3	1.4	2.5	4.5	12.4	16.6	12.0	7.0	17.6	17.3	17.9	8.5	6.1	2.4	6.0	1.5	5.1	5.7
UADBA 39003	M	35.4	3.3	4.6	3.1	1.3	2.0	3.8	11.6	15.1	11.1	6.8	17.7	19.6	20.6	8.7	5.2	2.0	5.9	1.2	5.7	6.1

[a]Holotype
[b]ID molecular

Table S4. Measurement data for *Mantidactylus ambony*. All measurements in mm. F: female, M: male, J: juvenile, SA: subadult

**Specimen**	**Sexes**	**SVL**	**TD**	**ED**	**EN**	**EST**	**NS**	**NN**	**HW**	**HL**	**HDL**	**RC**	**FE**	**TI**	**FT**	**TA**	**EO**	**EM**	**EHEAD**	**T1**	**TO3**	**TO5**
UADBA 7226	SA	30.3	3.4	4.8	3.2	1.7	2.0	3.6	11.4	14.5	10.2	6.9	17.6	19.9	18.0	9.0	6.7	1.9	5.3	1.4	4.4	5.2
UADBA 7225	F	35.3	2.2	4.0	3.6	1.5	2.0	3.6	11.3	14.8	10.7	6.3	18.3	19.3	19.0	9.0	6.4	2.0	5.0	1.3	4.8	5.2
UADBA 9058	F	34.0	2.9	4.9	3.1	1.4	1.1	3.1	11.8	13.0	9.7	7.3	18.0	19.3	18.5	9.6	6.1	2.2	5.6	1.2	5.2	5.5
AMNH A167487	F	36.8	2.9	4.2	2.4	1.6	1.9	3.1	10.7	14.1	11.6	6.3	19.0	20.3	19.7	8.7	6.3	2.0	5.3	1.2	5.3	6.0
AMNH A167491	F	37.1	3.3	4.1	2.1	1.3	1.5	3.4	11.2	15.0	11.7	7.8	18.7	21.1	19.0	9.5	5.6	2.0	5.3	1.7	5.5	6.1
AMNH A167493	F	37.9	2.7	4.3	2.7	1.9	1.6	3.3	10.8	13.8	11.0	7.4	19.4	21.5	20.3	9.8	5.6	2.3	5.6	1.2	5.0	5.6
AMNH A167494	F	37.7	3.1	4.1	2.7	1.8	1.8	2.9	11.5	15.5	10.4	7.7	20.2	20.8	18.8	10.1	6.4	2.4	5.2	1.1	5.1	6.1
AMNH A167495	F	36.7	2.7	4.5	2.5	1.6	1.8	3.4	10.6	13.4	10.7	7.6	19.0	20.3	18.7	9.0	6.0	1.9	5.0	1.3	5.1	5.3
AMNH A167498	F	37.2	3.3	4.5	2.6	1.8	2.0	3.4	11.4	15.1	10.7	6.4	19.2	20.1	19.8	9.1	5.6	2.0	5.3	1.2	5.1	6.4
AMNH A167490	M	30.0	4.4	3.8	2.3	1.5	1.6	2.6	9.4	13.1	9.2	5.7	16.0	16.3	15.5	7.8	5.2	1.8	4.8	1.1	4.1	4.7
AMNH A167492	M	31.3	4.7	3.5	2.6	1.5	1.5	2.8	9.0	14.6	8.6	6.0	15.1	16.7	16.3	7.6	5.1	1.7	4.4	0.8	4.4	5.0
AMNH A167497	M	31.0	4.9	3.8	2.3	1.3	1.5	2.6	9.4	13.5	9.5	5.6	15.4	17.2	16.8	7.8	5.3	1.9	5.0	1.1	4.6	5.4
UADBA 5726	M	30.6	4.0	4.3	2.4	1.2	1.4	3.0	10.5	12.8	9.8	5.6	15.4	17.1	15.5	7.0	4.6	1.8	4.4	1.2	4.5	4.9
UADBA 8406	M	31.8	5.0	4.2	2.8	1.7	2.0	3.3	11.0	14.8	9.9	6.2	14.4	16.0	15.3	8.0	5.7	1.6	4.5	1.4	3.7	4.3

Table S5. Measurement data for *Mantidactylus mocquardi*. All measurements in mm. F: female, M: male, J: juvenile, SA: subadult

**Specimen**	**Sexes**	**SVL**	**TD**	**ED**	**EN**	**EST**	**NS**	**NN**	**HW**	**HL**	**HDL**	**RC**	**FE**	**TI**	**FT**	**TA**	**EO**	**EM**	**EHEAD**	**T1**	**TO3**	**TO5**
AMNH A157111	F	46.0	2.6	5.7	3.9	2.2	2.3	3.9	16.6	20.4	14.4	9.3	24.2	24.0	24.5	9.5	8.4	2.9	6.8	[illegible]	5.5	6.5
AMNH A157113	F	51.4	3.1	8.0	4.5	2.6	2.2	4.7	17.5	23.0	15.4	9.9	26.1	26.9	26.6	11.2	8.9	3.6	8.6	2.5	6.5	8.0
AMNH A157116	F	51.0	3.6	7.2	4.4	2.3	1.9	4.8	16.8	21.6	14.4	9.6	26.7	25.8	25.1	10.2	8.6	3.2	7.9	2.3	6.4	7.5

AMNH A157119	F	50.9	2.9	6.6	4.2	2.0	2.5	4.1	16.5	21.5	13.4	8.6	25.7	25.3	24.3	9.9	8.3	2.7	7.1	1.6	7.3	7.8
AMNH A167583	F	51.9	3.6	6.4	4.5	3.3	2.7	3.6	18.1	23.1	15.3	9.7	28.8	26.9	25.0	11.6	9.0	3.4	8.6	2.2	6.5	7.7
AMNH A167587[b]	F	51.8	3.7	6.4	4.5	2.8	2.7	3.9	17.2	22.1	15.2	9.4	26.0	25.6	25.0	10.3	9.5	3.2	7.7	2.4	6.1	7.0
AMNH A 167589	F	52.4	4.3	6.4	4.8	2.05	2.0	4.8	18.4	21.6	15.3	9.2	28.2	26	27.7	10.6	9.6	3.8	8.6	2.7	7.2	8.5
AMNH A174621[b]	F	51.1	3.3	8.0	4.8	2.4	2.5	5.7	18.1	22.7	15.4	9.4	26.8	26.7	26.0	10.5	9.3	3.3	7.7	2.3	6.8	7.8
UADBA7229	F	64.3	5.2	8.8	5.6	1.3	2.5	6.6	23.4	26.7	19.6	12.0	33.1	35.4	33.7	14.6	11.9	4.3	10.8	2.5	8.8	10.4
UADBA7765	F	50.9	3.5	6.4	4.7	2.6	3.0	5.0	17.3	22.5	15.3	9.1	27.0	26.5	25.2	11.0	9.0	2.8	7.1	2.2	6.9	7.5
UADBA7767	F	51.8	3.6	8.6	4.5	3.1	3.5	5.6	18.3	23.8	14.8	8.9	27.5	27.1	25.4	12.2	7.6	2.8	7.9	1.7	6.2	7.1
UADBA7769	F	49.9	3.3	6.8	4.2	2.8	3.5	4.5	17.6	20.9	14.5	8.5	25.4	24.9	23.1	11.0	9.6	3.2	7.8	1.8	6.5	7.1
UADBA7772	F	49.0	3.2	7.0	3.7	2.4	3.0	3.0	16.8	22.2	14.0	9.2	25.4	25.4	25.4	11.1	8.0	2.5	6.9	2.3	5.9	7.4
UADBA7773	F	50.6	3.0	6.7	4.5	2.6	3.0	4.1	16.9	21.5	16.3	9.1	26.6	27.2	27.0	11.0	7.5	2.8	8.1	2.1	6.1	7.7
UADBA7777	F	47.5	3.1	6.9	4.2	2.7	2.8	4.6	15.8	20.6	13.0	9.0	24.4	25.2	24.8	10.2	7.8	2.6	6.9	2.0	6.1	6.9
UADBA 8118	F	50.0	3.7	7.0	4.2	2.3	3.6	5.0	17.7	22.6	14.6	9.5	26.1	25.5	24.3	11.0	8.4	2.5	7.5	2.4	6.9	7.4
UADBA8119	F	52.5	3.4	6.7	4.3	2.1	3.3	5.0	17.9	22.4	15.1	9.0	27.7	27.6	26.2	10.6	8.4	2.6	7.7	2.0	6.9	7.8
UADBA8124	F	61.8	4.3	7.9	5.3	3.0	4.3	6.4	22.5	28.4	19.0	11.1	31.6	31.5	31.5	13.1	9.0	4.0	10.2	2.5	7.6	8.8
UADBA 12312	F	56.0	4.0	6.6	4.9	2.8	2.8	5.0	18.0	25.0	16.0	10.0	27.2	28.1	28.2	11.9	9.6	3.4	8.4	2.1	6.8	8.4
UADBA 12313	F	52.8	4.0	7.0	4.7	3.1	3.8	5.4	18.6	24.3	15.2	11.0	29.6	29.3	29.4	12.8	9.3	3.0	8.4	2.5	8.3	9.3
UADBA 12315	F	55.6	4.3	7.7	5.0	2.8	3.6	5.3	19.0	24.9	16.0	9.6	29.1	29.0	28.0	11.5	8.8	3.3	8.4	2.7	7.5	8.3
UADBA 19603	F	48.8	3.9	6.4	3.7	2.5	2.4	4.0	16.8	20.8	11.4	9.7	24.8	25.3	25.1	11.4	8.4	3.0	7.4	2.3	7.0	7.6
UADBA 19609	F	55.2	3.3	7.0	4.7	2.9	3.0	4.6	17.4	24.5	16.1	11.0	28.3	28.2	27.4	11.3	9.5	3.3	7.6	2.3	8.0	8.5
UADBA 19610	F	57.5	3.4	8.1	4.8	2.9	2.6	4.7	18.5	24.3	17.2	10.4	30.1	29.8	29.3	12.7	8.6	3.3	9.1	2.3	8.3	8.5
UADBA 19646	F	53.4	3.2	7.0	4.3	2.8	3.3	4.7	16.8	22.5	16.4	11.0	28.5	28.2	28.3	11.8	9.2	3.0	7.5	2.4	7.8	8.5
UADBA 19647[b]	F	59.1	3.6	7.6	5.0	3.0	3.5	4.7	17.1	24.4	16.1	10.0	28.1	27.4	27.2	11.8	9.3	3.2	8.5	2.2	6.5	8.0
UADBA 19649	F	53.3	3.4	8.2	5.1	2.4	3.3	4.8	18.1	23.6	16.7	9.6	28.5	27.3	28.4	12.3	8.2	2.6	7.6	2.4	7.4	8.5
UADBA19686	F	51.0	4.3	6.9	4.7	2.7	3.3	5.0	17.6	22.3	15.2	9.6	27.0	27.0	26.7	12.0	8.7	3.0	8.3	2.2	7.1	7.6
UADBA19687	F	57.8	4.8	8.1	5.1	2.9	3.1	5.3	19.4	24.0	14.3	9.9	28.3	28.1	26.2	12.0	10.2	3.2	8.0	2.3	7.1	8.2
UADBA 26232	F	51.4	3.4	6.0	4.5	2.0	3.1	4.2	18.3	22.3	19.6	9.8	29.1	27.7	26.5	12.8	9.3	2.8	8.6	2.1	7.1	8.5
UADBA 26233	F	51.2	3.9	7.6	3.6	3.0	3.1	4.4	16.8	21.9	13.8	9.6	26.3	25.5	26.5	11.0	8.2	2.8	8.6	2.6	6.9	7.5
UADBA 26234	F	52.2	3.1	8.0	4.2	2.4	3.2	4.7	17.8	21.0	14.6	7.7	26.7	26.1	25.3	11.4	9.1	3.2	8.2	2.2	7.2	8.0
UADBA 26235	F	52.0	4.4	7.6	3.1	2.8	1.2	5.5	20.0	23.7	17.0	10.7	30.3	30.0	28.5	13.7	10.1	3.3	8.4	2.4	7.6	8.6
UADBA 26236	F	55.3	4.1	5.8	4.5	3.1	3.6	4.2	18.0	25.2	15.7	9.5	29.1	28.5	28.5	11.9	9.2	3.1	8.4	2.4	7.1	8.6
UADBA 26237	F	49.8	3.8	6.5	4.1	2.8	3.0	4.0	17.1	20.4	15.0	9.7	26.7	27.5	25.5	12.1	10.0	3.4	8.4	2.2	6.5	7.0
UADBA 26238	F	53.4	3.8	6.6	4.5	2.7	2.3	4.3	17.8	23.4	14.4	9.6	27.5	27.1	26.6	12.6	9.5	3.0	8.6	2.0	7.1	8.0

UADBA 26239	F	54.1	3.5	6.7	3.8	2.6	3.1	4.9	16.9	22.4	14.4	9.2	27.7	26.9	27.2	11.5	8.6	3.3	7.7	2.0	6.7	7.5
UADBA 26240	F	59.3	4.6	8.6	5.1	3.5	4.1	6.0	20.2	27.6	16.9	10.3	29.3	29.3	28.0	12.0	10.7	3.7	9.3	2.4	7.1	8.0
UADBA 26241	F	50.7	3.7	6.4	4.1	3.0	2.8	4.8	18.5	22.7	16.0	9.9	28.5	28.3	26.8	10.7	9.1	4.1	7.4	2.0	7.0	8.6
UADBA 26242	F	53.3	4.3	7.7	4.8	2.8	4.0	5.9	19.3	23.6	17.0	9.8	28.3	28.5	28.4	12.2	8.5	2.9	7.5	2.1	7.8	8.3
UADBA 26243	F	49.0	3.3	6.4	3.3	2.7	3.2	4.7	16.4	19.7	15.1	9.7	26.9	27.0	25.0	12.3	8.2	3.0	7.3	2.0	7.3	8.4
UADBA 26245	F	50.1	3.1	6.4	4.4	3.1	3.5	5.0	19.2	23.8	16.1	9.8	29.4	27.3	26.6	11.4	8.8	2.2	7.4	2.0	6.7	7.5
UADBA 26246	F	50.6	3.5	7.0	3.8	2.8	3.1	4.3	16.1	21.3	15.8	9.4	27.1	26.6	26.2	12.6	8.8	3.0	7.5	1.9	6.8	7.8
UADBA 26247	F	53.4	3.7	6.3	4.2	2.9	2.4	4.0	17.3	22.3	15.2	10.9	28.9	27.2	26.7	11.4	9.1	3.3	8.0	2.0	6.5	7.0
UADBA 26248	F	53.3	3.1	6.0	4.7	2.8	2.1	3.8	18.1	22.4	15.4	10.2	27.7	27.2	27.5	11.2	9.1	3.1	7.8	2.0	7.0	7.8
UADBA 26410	F	53.8	4.3	7.0	5.4	3.3	3.6	5.3	18.7	23.9	16.1	10.7	28.4	29.1	26.4	12.1	9.0	3.7	8.6	1.8	7.2	8.6
AMNH A157118	M	40.2	3.8	5.8	3.0	1.9	2.0	3.2	13.0	18.6	12.0	7.0	20.0	29.6	20.1	7.8	7.4	2.2	5.7	1.5	5.3	5.7
AMNH A157120	M	37.6	3.6	5.3	2.7	2.1	2.3	3.0	11.6	16.0	11.0	7.2	19.7	19.0	19.0	7.7	6.0	2.4	5.4	1.5	5.0	5.2
AMNH A167584	M	44.9	3.8	4.4	3.8	2.3	2.7	3.4	14.6	21.2	13.7	8.3	24.4	22.3	22.5	9.1	8.2	2.8	7.1	1.8	6.0	7.0
AMNH A167585	M	39.0	3.9	5.1	3.3	2.2	2.3	3.8	12.8	18.0	12.3	7.2	21.3	20.1	20.8	9.0	7.0	2.2	5.8	1.9	5.2	6.1
AMNH A167586	M	40.7	4.4	5.7	3.4	2.7	2.8	3.0	13.6	19.2	12.0	7.5	21.4	19.4	20.0	8.3	7.6	2.6	6.7	2.0	5.5	5.7
AMNH A167597	M	38.8	5.1	5.1	3.0	2.1	2.3	3.6	13.8	18.1	12.2	6.1	21.5	20.0	20.4	8.8	7.3	2.2	6.2	1.7	5.1	6.2
AMNH A174622[b]	M	40.5	4.8	5.6	3.1	2.0	2.6	3.8	13.0	19.1	12.4	6.7	19.8	20.0	20.0	7.7	7.3	2.9	6.0	1.6	5.3	5.5
AMNH A174628[b]	M	41.5	5.1	5.9	3.0	2.1	2.2	3.2	13.7	20.5	12.1	7.0	20.2	20.2	21.5	8.9	6.8	2.4	6.9	1.8	4.8	6.6
UADBA 3719	M	40.5	5.2	5.8	3.6	2.6	2.7	4.0	13.8	18.1	12.3	7.7	20.6	20.0	20.3	10.0	6.5	2.3	6.8	1.7	5.4	6.0
UADBA 6282	M	39.5	4.3	5.9	3.2	2.2	2.7	4.0	13.1	18.1	11.2	7.3	19.6	19.5	19.4	8.3	6.2	2.1	5.6	1.3	4.7	5.4
UADBA 7312	M	44.0	4.3	5.9	3.6	1.9	2.7	4.5	13.4	18.8	12.9	8.7	21.2	21.4	20.6	8.5	6.6	2.3	6.6	1.5	5.5	6.1
UADBA 7313	M	36.7	3.8	4.5	3.6	2.1	2.4	3.8	12.4	16.5	11.3	8.8	20.4	20.0	19.0	9.7	6.8	2.1	6.0	1.6	5.3	6.1
UADBA 7774	M	37.0	4.2	5.8	3.2	2.1	2.0	3.8	12.3	16.7	11.1	6.6	20.7	19.4	19.1	8.3	6.2	2.5	5.6	1.5	5.2	5.8
UADBA 7766	M	40.5	4.1	6.1	3.6	2.2	2.6	3.8	13.1	17.1	11.3	5.8	21.4	19.6	19.5	7.3	6.9	2.1	5.7	1.6	4.8	5.6
UADBA 8121	M	43.3	5.2	6.5	3.4	2.5	3.1	4.4	15.8	18.7	12.1	7.6	22.6	21.1	21.0	9.2	6.7	2.2	5.6	1.6	5.2	5.6
UADBA 12310	M	38.7	4.0	4.2	3.4	2.1	1.4	3.0	12.4	17.9	11.3	6.1	20.0	20.1	17.6	9.2	6.2	2.0	6.1	1.6	5.2	6.2
UADBA 12311	M	38.4	4.0	4.6	3.2	2.1	2.0	3.4	11.6	19.1	11.0	7.6	19.4	20.2	20.6	8.3	6.4	2.1	6.4	1.2	5.5	6.2
UADBA 19600	M	40.4	3.7	5.6	3.8	2.2	2.6	3.4	12.7	19.0	12.4	6.9	21.6	20.8	20.1	8.8	7.6	2.2	5.6	1.7	5.9	6.3
UADBA 19601	M	37.4	3.2	4.8	3.8	2.4	2.6	3.4	12.9	16.8	12.1	7.0	19.8	19.6	18.9	9.0	7.0	2.1	5.3	1.5	5.4	5.7
UADBA 19602	M	45.3	4.1	6.4	4.1	2.6	3.7	4.1	14.4	21.2	12.9	7.8	23.4	21.8	22.7	9.6	7.0	2.3	6.5	1.9	5.7	6.7
UADBA 19604	M	39.7	5.0	5.3	3.4	2.1	2.6	3.7	12.9	19.0	12.4	7.6	20.4	20.0	20.3	9.2	9.4	3.6	9.0	1.7	7.0	7.6
UADBA 19605	M	39.8	5.2	5.2	3.2	1.9	1.2	3.6	12.8	18.2	12.4	7.2	21.0	19.7	19.8	8.8	6.8	2.4	6.5	1.5	5.0	5.9
UADBA 19606	M	41.3	4.0	5.3	3.8	2.4	2.4	3.6	13.3	18.8	12.2	7.5	21.2	20.9	21.7	9.2	6.9	2.1	5.7	1.7	5.0	5.8

UADBA 19596	M	39.9	4.2	5.3	3.7	2.3	2.3	3.4	12.4	18.9	11.6	7.0	20.1	19.5	19.7	9.1	7.1	2.3	6.4	1.6	5.2	6.1
UADBA 19685	M	36.1	4.0	5.4	2.9	2.0	2.7	4.2	11.9	17.7	11.1	5.6	18.3	18.3	18.0	7.8	6.4	2.0	5.7	1.3	4.8	5.3
UADBA 19689	M	38.4	4.4	5.0	3.3	2.1	2.1	4.6	13.1	16.8	11.4	7.6	20.8	20.6	20.5	9.1	7.1	2.3	6.0	1.5	5.6	6.2
UADBA 19690	M	36.6	4.1	5.3	3.2	2.0	2.5	4.0	12.5	16.1	10.4	6.3	19.0	19.3	18.2	7.3	6.3	2.3	5.5	1.3	4.8	5.6
UADBA 26298	M	42.0	4.7	5.3	4.0	2.6	3.1	4.6	14.2	19.0	12.8	7.8	20.2	20.2	21.0	9.6	7.5	2.4	6.3	1.3	5.0	6.1
UADBA 26300	M	43.1	5.2	6.3	3.6	2.4	3.2	4.3	15.2	19.3	12.1	7.3	23.0	21.2	21.0	8.3	7.9	2.6	6.3	1.6	5.0	6.0
UADBA 26303	M	38.4	4.0	5.5	3.4	2.1	2.0	3.3	11.9	16.4	11.6	7.3	21.7	20.4	20.3	8.5	6.3	2.0	5.4	1.6	5.6	6.0
UADBA 26304	M	36.3	3.9	4.8	3.3	2.1	2.4	3.1	11.6	16.7	12.1	7.7	20.3	19.4	20.0	8.4	6.4	2.0	5.2	1.3	5.0	5.4
UADBA 26305	M	38.2	3.5	5.0	3.6	2.1	2.6	3.5	11.6	17.3	11.2	6.5	18.8	18.1	18.8	8.1	6.8	2.1	5.3	1.5	4.4	5.3
UADBA 19640	M	36.6	5.0	5.0	3.2	2.1	2.3	3.3	12.6	17.5	11.5	6.8	20.8	19.2	18.6	7.8	5.9	1.8	5.5	1.3	4.7	5.2
UADBA 19648	M	41.2	4.2	5.6	2.9	2.2	2.4	3.6	13.0	17.2	11.8	6.6	18.8	19.8	19.4	8.2	7.1	2.2	6.5	1.6	5.4	5.8
UADBA 19652	M	44.2	4.8	6.3	3.4	2.3	2.8	4.2	14.6	20.5	13.0	7.0	21.7	20.6	21.0	7.9	7.6	2.2	6.6	1.7	4.9	5.4
UADBA 19653	M	38.4	4.0	5.5	3.4	2.1	2.0	3.3	11.9	16.4	11.6	7.3	21.7	20.4	20.3	8.5	7.5	2.9	6.0	2.1	5.6	6.2
UADBA 19654	M	40.8	4.4	5.7	3.6	2.1	2.3	3.8	13.0	17.6	12.6	7.7	20.2	21.0	21.3	8.8	7.6	2.2	6.0	1.7	6.1	6.5
UADBA 19597	M	39.1	4.1	5.8	3.6	2.0	2.7	3.8	13.2	19.2	12.9	7.5	21.4	20.8	20.5	9.8	7.3	2.4	5.8	2.1	5.0	6.2
UADBA 26279	M	43.2	4.8	6.1	3.3	2.5	3.2	3.7	15.2	20.3	12.4	8.3	21.5	21.7	21.6	9.0	6.8	2.4	7.3	1.8	5.4	6.2
UADBA 26285	M	40.4	4.0	5.4	3.7	2.0	2.1	3.3	13.1	18.4	11.4	7.0	21.2	20.6	20.0	7.8	7.1	2.4	6.0	1.7	5.3	6.0
UADBA 26286	M	41.8	4.4	6.3	3.5	2.5	2.9	3.7	14.1	19.3	12.4	6.6	21.8	19.5	21.7	8.3	6.9	2.6	6.5	1.7	5.3	5.6
UADBA 26287	M	44.7	4.3	5.0	3.6	2.3	2.8	3.4	14.5	18.6	13.2	7.7	23.3	22.9	23.8	9.9	7.7	2.5	6.7	1.7	5.7	7.0
UADBA 26288	M	40.0	4.0	5.2	3.3	1.8	2.3	3.7	12.7	16.7	11.6	7.1	20.0	20.2	18.8	9.0	5.6	2.5	5.8	1.4	5.4	6.1
UADBA 26289	M	40.6	3.8	5.2	2.9	2.0	2.2	3.8	13.1	18.1	12.2	7.3	21.1	20.1	21.0	8.7	6.9	2.1	5.8	1.6	5.0	6.2
UADBA 26290	M	39.5	4.3	4.6	3.1	2.2	3.1	3.1	13.4	18.0	12.4	7.2	21.3	20.9	21.4	9.1	6.0	2.3	6.4	1.7	5.6	6.2
UADBA 26291	M	38.6	3.5	5.0	2.7	2.0	2.4	3.4	13.6	16.1	12.2	7.5	20.4	20.0	20.2	8.4	6.1	2.3	6.0	1.6	5.4	5.8
UADBA 26292	M	40.1	3.4	4.5	3.1	2.5	2.5	3.7	13.4	18.1	11.9	6.6	20.7	20.6	20.5	8.6	6.6	2.4	6.1	1.9	5.5	5.9
UADBA 26293	M	42.7	4.0	5.9	3.1	2.4	2.6	3.6	13.1	19.5	12.7	6.5	21.6	20.1	21.6	9.4	6.7	2.5	6.8	1.5	5.2	6.0
UADBA 26299	M	36.1	3.7	4.9	3.0	2.3	2.6	3.9	12.4	16.5	11.3	6.7	19.5	19.0	18.6	8.6	6.8	2.2	6.4	1.7	4.9	5.1

[b] ID molecular

Table S6. Measurement data for *Mantidactylus zolitschka*. All measurements in mm. ZFMK specimen data from Glaw and Vences (2004). F: female, M: male, J: juvenile, SA: subadult

Specimen	Sexes	SVL	TD	ED	EN	EST	NS	NN	HW	HL	HDL	RC	FE	TI	FT	TA	EO	EM	EHEAD	T1	TO3	TO5
UADBA 6965	M	26.5	2.8	3.6	1.9	0.6	1.3	1.6	8.8	11.2	8.0	5.2	14.3	14.5	15.4	6.8	4.4	2.0	5.4	0.8	4.8	5.3
UADBA 6966	M	27.7	3.0	3.6	2.0	1.2	1.4	2.3	9.4	13.1	9.0	5.3	14.8	14.7	15.5	6.2	4.6	1.9	4.9	0.9	4.8	5.3

ZFMK 60110[a]	M	30.6	3.0	3.7	2.7	-	1.8	3.3	10.5	12.0	10.0	-	-	-	16.9	-	-	-	-	-	-
ZFMK 60112	M	28.8	3.2	3.7	2.5	-	1.8	3.0	9.9	11.3	10.0	-	-	-	15.2	-	-	-	-	-	-
ZFMK 60113	M	29.8	3.1	3.3	2.7	-	2.0	3.1	10.0	11.7	9.9	-	-	-	16.2	-	-	-	-	-	-
ZFMK 60114	M	30.6	3.0	3.3	2.5	-	1.8	2.8	10.0	11.6	9.6	-	-	-	16.7	-	-	-	-	-	-
ZSM 939/2000	M	29.6	3.2	3.4	2.4	-	1.8	2.6	10.0	11.4	10.2	-	-	-	17.2	-	-	-	-	-	-
ZFMK 60115	F	37.7	2.8	4.5	3.5	-	2.2	3.7	12.8	15.0	11.9	-	-	-	20.9	-	-	-	-	-	-
ZFMK 60116	F	37.6	3.0	4.3	3.3	-	2.4	3.6	12.8	14.3	12.0	-	-	-	20.8	-	-	-	-	-	-
ZSM 184/2003	F	33.6	2.5	4.2	3.4	-	2.3	2.9	11.3	14.0	10.6	-	-	-	19.5	-	-	-	-	-	-

[a]Holotype

Table S7. Measurement data for *Mantidactylus catalai*. All measurements in mm. F: female, M: male, J: juvenile, SA: subadult

Specimen	Sexes	SVL	TD	ED	EN	EST	NS	NN	HW	HL	HDL	RC	FE	TI	FT	TA	EO	EM	EHEAD	T1	TO3	TO5
AMNH A133690	F	58.9	3.5	6.9	3.6	2.0	1.3	5.3	19.3	22.7	17.2	9.7	29.3	31.2	30.3	13.8	10.0	3.2	8.3	2.0	8.2	9.3
UADBA3706	F	52.1	4.1	6.2	4.7	2.3	2.4	4.8	17.1	22.2	13.0	9.8	27.0	29.2	26.8	11.3	10.1	2.8	7.6	2.3	7.2	8.0
UADBA4513	F	53.6	4.1	6.0	4.6	2.1	2.3	4.9	18.3	22.8	17.0	9.2	28.0	28.3	27.7	12.0	8.8	2.9	6.7	2.4	7.1	8.2
UADBA9772	F	61.5	4.5	6.5	4.8	1.6	2.8	5.1	19.4	26.6	16.7	9.6	29.5	29.8	29.4	14.0	9.7	3.7	8.1	2.0	7.6	8.6
UADBA9773	F	52.7	4.0	6.2	4.7	2.8	2.2	5.1	18.1	22.5	15.7	9.8	28.1	28.1	28.3	12.4	8.8	3.6	7.5	1.8	7.3	8.3
UADBA9774	F	60.0	4.1	8.2	5.4	3.5	3.8	5.6	21.0	25.9	18.1	10.7	29.6	30.9	30.5	13.4	10.4	3.3	9.1	2.4	8.2	9.1
UADBA26403	F	51.9	4.3	7.8	4.4	3.0	2.8	5.8	20.8	25.4	18.0	11.3	31.8	32.1	32.0	14.0	10.3	3.5	9.3	2.5	7.7	8.6
UADBA26404	F	57.9	4.2	7.4	5.4	2.4	3.6	4.8	19.3	24.3	16.9	11.2	30.1	29.2	30.0	12.6	10.0	3.6	8.6	2.4	8.8	9.7
UADBA26405	F	57.0	4.5	7.7	4.7	2.6	1.5	5.0	18.7	23.8	16.9	11.8	31.0	30.7	29.4	14.2	9.3	3.5	8.4	2.5	8.1	8.5
UMMZ 212890[b]	F	63.3	4.7	7.5	4.9	2.9	3.3	4.7	21.5	27.5	17.9	12.1	31.9	32.0	30.0	13.5	11.3	4.1	8.6	3.0	8.5	9.1
AMNH A168364	M	42.3	4.7	5.6	3.3	2.2	2.2	3.7	18.5	19.3	13.1	9.2	21.6	20.7	20.2	9.4	6.8	3.1	6.8	1.8	5.6	6.1
UADBA1419	M	42.6	6.2	6.0	3.8	2.6	2.4	4.0	15.0	21.1	17.9	8.1	22.5	22.5	23.0	10.3	7.3	2.3	7.7	2.2	6.1	6.4
UADBA1420	M	41.1	5.1	5.1	3.4	2.6	2.3	4.5	13.8	17.8	12.0	7.9	20.6	22.4	20.1	8.4	6.6	2.4	7.3	1.8	5.3	5.8
UADBA1421	M	44.6	5.8	5.6	3.6	2.8	2.7	4.8	15.0	20.2	13.6	9.6	22.4	23.2	22.1	11.1	7.7	2.0	7.5	1.6	6.1	6.6
UADBA1423	M	43.9	5.0	5.0	3.9	2.3	1.9	4.4	15.3	20.0	13.8	8.4	21.8	23.6	24.5	10.1	7.2	2.9	7.0	2.0	6.4	7.2
UADBA4514	M	45.4	5.1	5.1	4.1	1.8	2.7	4.3	15.0	20.6	14.1	8.6	24.0	23.5	23.3	10.6	7.4	2.4	7.1	1.8	5.8	6.2
UADBA4516	M	43.9	5.1	5.1	3.7	2.6	3.3	4.9	14.5	19.0	13.5	8.0	22.1	22.0	21.8	11.2	6.8	2.4	7.4	1.8	5.9	6.2
UADBA4521	M	45.4	5.2	5.8	4.3	3.2	2.6	4.3	14.8	19.6	15.0	12.7	22.8	22.6	22.2	9.7	7.0	2.7	7.3	1.9	6.1	6.8
UADBA4522	M	45.2	6.6	6.2	4.3	2.8	2.7	4.1	15.3	20.4	12.8	8.0	21.3	21.2	20.8	10.0	7.4	2.3	6.7	2.1	5.8	6.1
UADBA4523	M	43.0	5.6	5.2	3.6	3.1	2.1	4.3	15.0	19.0	12.7	8.0	22.0	21.4	20.8	11.4	6.9	2.6	7.3	2.3	5.4	6.0
UADBA9782	M	43.4	5.0	5.2	3.5	3.1	2.4	3.4	14.7	18.5	13.0	8.2	22.6	21.6	20.7	11.5	6.7	2.6	6.3	1.8	6.0	6.4

[b]ID Molecular

Table S8. Measurement data for *Mantidactylus poissoni*. All measurements in mm. F: female, M: male, J: juvenile, SA: subadult

Specimen	Sexes	SVL	TD	ED	EN	EST	NS	NN	HW	HL	HDL	RC	FE	TI	FT	TA	EO	EM	EHEAD	T1	TO3	TO5
MNHN 1937.1*	F	53.4	3.4	6.4	4.3	1.6	2.8	4.8	16.1	21.2	13.5	9.4	29.1	30.8	29.1	13.6	9.5	3.1	7.7	1.7	7.2	8.5
AMNH A174653*	-	-	-	-	-	-	-	-	-	-	-	-	-	-	-	-	-	-	-	-	-	-
AMNH A174650	F	64.4	3.3	6.0	5.4	2.4	3.5	5.6	21.8	27.2	20.2	13.7	34.5	37.3	35.2	15.9	10.5	3.5	10.8	3.1	9.2	10.8
UADBA 6876	F	55	4.2	6.6	5.0	2.7	2.9	5.0	19.4	23.8	17.6	11.9	31.8	31.8	31.3	13.7	9.7	3.4	8.7	2.4	9.0	9.6
UADBA 7125	F	65.3	4.0	9.0	5.4	2.3	3.5	5.9	22.1	26.9	18.4	13.0	35.7	35.4	32.0	16.1	12	3.4	10.0	2.3	9.0	10.2
UADBA 19786	SA	51.0	4.2	7.2	4.3	2.0	2.8	5.8	17.4	24.2	15.3	9.4	26.9	27.3	27.3	11.0	8.5	3.1	8.2	1.5	7.3	8.4
UADBA 26409	F	63.9	4.7	7.1	5.3	3.3	4.6	6.0	20.5	27.8	17.5	11.1	30.0	30.3	30.2	12.0	11.4	3.8	10.2	2.8	11.0	11.4
UADBA 26411	F	60.8	4.3	7.6	5.3	2.7	2.9	4.9	22.2	25.9	18.0	11.8	33.3	34.9	32.5	15.5	10.3	3.4	9.7	2.9	9.0	10.0
UADBA 26412	F	59.4	4.5	6.5	4.1	2.0	2.1	4.6	20.7	24.2	20.0	11.4	30.5	33.3	34.3	15.1	9.8	3.4	9.3	2.7	9.4	10.3
AMNH A50362	M	39,7	3.6	5.8	3.6	1.5	1.8	4.0	12.5	17.7	13.0	9.0	–	–	–	–	7.4	2.7	6.8	–	–	–
UADBA 11899	M	48.2	5.7	6.5	4.2	2.4	3.7	5.0	17.1	22.2	14.6	10.0	24.8	26.0	26.6	11.0	9.2	2.9	7.6	1.8	7.3	8.3
UADBA 39000	M	46.8.	6.0	6.1	3.8	2.8	2.1	4.3	16.8	21.0	13.3	9.6	24.4	24.3	22.7	8.8	10.0	4.0	9.4	2.0	7.6	8.6

*Reference specimen

Table S9. Measurement data for *Mantidactylus danieli* n. sp.. All measurements in mm. F: female, M: male, J: juvenile, SA: subadult

Specimen	Sexes	SVL	TD	ED	EN	EST	NS	NN	HW	HL	HDL	RC	FE	TI	FT	TA	EO	EM	EHEAD	T1	TO3	TO5
AMNH A167523[b]	J	27.8	-	-	-	-	-	-	-	-	-	-	-	-	-	-	-	-	-	-	-	-
AMNH A167578	F	53.0	6.5	9.4	4.7	0.6	1.1	3.8	20.4	25.1	14.9	11.7	26.0	27.4	29.7	13.6	9.4	3.3	8.8	1.9	7.6	8.7
AMNH A167579	F	47.7	4.8	8.7	4.2	0.4	1.6	4.0	17.4	20.0	14.4	9.6	23.6	25.7	25.9	12.5	8.7	3.3	7.7	2.2	6.9	7.2
AMNH A167590*[b]	F	50.6	3.8	5.1	2.4	1.0	1.9	3.6	17.4	20.6	13.1	9.2	24.1	24.7	25.2	11.5	8.8	3.0	7.6	1.9	6.2	7.4
AMNH A167592	F	46.1	3.7	4.9	2.0	1.5	2.5	4.1	16.8	20.2	13.6	8.5	24.9	25.8	25.9	11.5	8.3	3.0	7.4	1.8	6.3	7.0
UADBA 8382	F	53.0	4.6	6.0	4.7	1.9	3.3	5.0	18.4	24.3	14.3	10.7	25.3	27.2	26.7	14.5	9.5	3.1	8.9	2.3	6.1	7.4
UADBA 12140	F	42.3	3.6	5.0	4.0	2.1	2.3	4.3	15.5	19.0	12.7	8.1	22.1	23.4	23.5	11.0	7.5	2.7	6.5	1.7	6.1	7.0
UADBA 19593	F	50.1	3.5	6.1	4.3	1.4	2.3	3.6	16.3	21.6	11.0	9.6	26.1	28.1	28.9	14.3	8.5	2.5	7.6	1.8	7.7	8.7
UADBA 26365	F	49.0	4.4	6.6	3.4	2.1	2.9	4.6	16.8	20.9	14.5	10.3	25.7	27.4	28.0	12.4	8.2	3.0	7.7	1.8	6.7	7.8
UADBA 26366	F	48.8	4.3	6.3	4.0	2.3	3.0	4.7	16.7	22.1	15.5	8.5	25.5	27.1	28.2	11.9	8.8	2.8	7.7	2.0	6.4	7.6
UADBA 26367	F	49.4	4.1	6.0	4.0	2.6	2.7	4.8	16.7	21.8	15.1	10.4	26.1	27.1	27.8	13.0	9.2	2.9	7.9	1.7	6.2	7.6
UADBA 26368	F	48.4	4.1	5.8	4.2	2.4	2.1	4.3	16.6	21.4	14.5	8.7	25.1	26.6	27.5	12.6	8.5	3.1	7.4	2.1	6.6	7.2

AMNH A50222	M	33.9	3.3	4.0	2.5	1.2	1.3	3.4	11.9	16.0	10.8	6.8	16.5	18.8	20.0	8.9	5.8	2.3	5.9	1.0	4.9	5.8
AMNH A167577	M	41.7	5.1	5.4	4.1	1.4	2.3	3.4	15.9	19.3	12.2	8.3	21.3	21.9	21.9	10.8	8.4	3.0	7.0	1.8	5.6	6.3
AMNH A167591	M	33.9	4.2	4.8	2.9	1.2	2.0	2.8	11.8	15.5	9.6	6.4	17.1	17.3	18.7	7.2	6.3	2.1	5.7	1.1	4.5	4.7
UADBA 3711	M	38.8	4.0	4.9	3.3	1.0	2.0	4.4	13.6	16.4	10.5	7.6	19.7	19.5	20.2	9.5	6.4	2.2	6.6	1.3	5.5	6.2
UADBA 3712	M	38.4	5.1	4.6	3.5	1.3	1.4	3.9	13.1	16.9	11.5	7.2	18.8	19.4	20.6	9.6	6.1	2.1	6.0	0.9	5.4	5.6
UADBA 3713	M	35.0	4.2	4.5	3.1	1.3	1.6	3.7	12.4	16.2	10.8	9.3	16.7	19.3	19.6	9.1	5.8	2.4	6.8	1.3	5.6	5.8
UADBA 3716	M	36.0	3.5	4.5	3.0	1.3	2.3	3.8	12.5	16.7	11.6	7.3	18.6	20.0	20.0	9.4	5.4	1.7	5.5	1.3	5.1	6.6
UADBA 8376	M	42.4	4.7	5.8	3.5	2.0	2.1	4.3	14.3	18.8	12.1	7.6	20.1	20.3	20.7	8.6	7.3	2.4	7.2	1.6	5.8	6.1
UADBA 19595	M	37.3	4.2	5.0	3.3	1.6	1.7	3.3	12.0	16.5	10.0	6.2	16.6	18.0	17.0	8.5	5.8	2.0	5.5	1.5	4.3	5.1
UADBA 26359	M	42.1	3.4	5.1	3.2	1.9	2.2	4.4	15.0	17.8	14.0	7.6	21.7	23.0	24.8	10.8	6.5	2.5	6.3	1.7	6.6	7.5
UADBA 26364	M	41.4	3.4	4.7	3.0	2.0	2.2	3.6	14.6	18.8	13.3	9.3	23.3	25.0	25.6	12.1	7.3	2.6	6.8	1.6	6.5	7.3
UADBA 26369	M	41.0	3.6	5.0	3.9	1.0	1.1	4.0	15.4	18.0	14.0	8.6	24.0	26.0	28.1	11.7	7.5	2.8	5.9	1.9	6.7	8.3
UADBA 26370	M	41.0	3.7	5.1	3.3	1.8	2.5	4.1	14.8	18.7	13.7	8.2	23.0	24.7	25.7	12.5	7.0	2.6	6.1	1.3	6.6	7.7
UADBA 26371	M	34.0	3.6	5.1	3.6	1.6	1.8	3.4	11.7	16.4	11.5	7.0	15.8	18.3	19.6	7.6	5.9	2.1	5.4	1.3	4.6	5.6
UADBA 26373	M	34.2	4.2	5.3	3.1	1.7	2.3	3.3	12.5	16.7	10.7	6.4	18.5	18.9	20.4	9.0	6.0	1.9	5.3	1.1	4.4	5.6
UMMZ 212835	M	51.9	4.9	5.9	4.5	1.8	1.8	4.0	17.8	24.4	15.6	10.4	28.8	31.6	26.2	9.8	9.7	3.3	8.6	1.9	7.4	8.5

[b]ID Molecular
* Holotype

Table S10. Measurement data of *Mantidactylus macrotympanum* n. sp.. All measurements in mm. F: female, M: male, J: juvenile, SA: subadult

Specimen	SEX	SVL	TD	ED	EN	EST	NS	NN	HW	HL	HDL	RC	FE	TI	FT	TA	EO	EM	EHEAD	TI	TO3	TO5
AMNH A167598	M	62.1	7.9	7.2	5.0	2.2	3.0	5.7	20.9	25.8	18.8	11.4	32.2	33.5	31.7	14.3	11.3	3.5	9.8	2.6	8.8	10.3
AMNH A167599*[b]	M	61.0	8.0	8.6	4.6	2.2	2.5	4.8	20.7	26.8	19.8	10.7	34.8	34.3	32.3	13.9	10.1	3.4	10.2	3.0	9.4	10.9
UMMZ 201416[b]	M	61.9	8.3	8.3	5.3	2.0	2.5	5.1	21.7	26.6	19.2	11.8	33.0	32.8	32.6	13.7	10.9	4.2	10.1	2.9	9.4	10.1
UMMZ 201417	M	59.4	7.9	7.4	4.8	2.1	2.9	4.9	20.3	25.9	18.1	11.5	32.0	33.5	31.3	13.7	10.7	4.1	10.5	1.9	9.4	10.1
UMMZ 201418	M	57.9	7.6	7.3	5.1	2.4	2.8	4.5	20.5	26.7	17.3	12.1	29.4	31.2	30.5	13.8	10.5	3.6	9.0	2.3	8.7	10.1
UMMZ 213447[b]	M	-	-	-	-	-	-	-	-	-	-	-	-	-	-	-	-	-	-	-	-	-

[?b]ID Molecular,
* Holotype

Table S11. Measurement data for *Mantidactylus olgai* n sp.. All measurements in mm. F: female, M: male, J: juvenile, SA: subadult

Specimen	SEX	SVL	TD	ED	EN	EST	NS	NN	HW	HL	HDL	RC	FE	TI	FT	TA	EO	EM	EHEAD	T1	TO3	TO5
AMNH A167565[b]	J	26.5	-	-	-	-	-	-	-	-	-	-	-	-	-	-	-	-	-	-	-	-

AMNH 181726[b]	J	-	-	-	-	-	-	-	-	-	-	-	-	-	-	-	-	-	-	-	-	-
AMNH A167556	F	45.8	2.9	4.9	4.0	2.3	2.3	3.2	15.2	18.7	13.5	9.1	25.8	24.0	23.0	8.8	8.0	2.7	6.9	1.3	5.8	6.7
AMNH A167558	F	49.6	3.6	6.7	2.7	2.8	2.4	4.0	15.4	22.1	15.1	8.4	25.5	23.7	25.4	9.8	8.7	3.0	7.4	2.1	6.2	7.6
AMNH A167561	F	46.5	3.6	5.8	3.9	2.5	2.4	3.5	16.4	18.9	13.0	7.5	23.6	22.6	22.6	10.0	8.6	3.1	6.9	1.7	5.7	6.2
AMNH A167562	F	51.8	2.6	6.6	4.0	2.3	2.3	3.9	16.9	21.8	14.5	9.2	25.0	24.1	24.8	11.5	8.6	3.4	7.5	2.4	6.6	7.6
AMNH A167563	F	51.0	3.2	6.4	3.7	2.9	2.8	3.8	17.1	20.6	14.1	7.7	25.1	23.7	22.5	9.8	8.7	3.2	7.8	1.8	5.7	6.4
AMNH A167564	F	46.8	3.5	5.0	3.8	2.6	2.8	4.3	16.7	21.6	14.7	8.4	23.6	24.0	25.3	10.5	8.8	3.0	9.2	1.7	6.8	7.8
AMNH A167569	F	46.5	3.2	5.8	3.6	2.7	3.0	4.3	15.8	20.2	14.0	7.7	23.7	23.0	23.7	9.6	8.5	3.0	7.8	1.5	6.3	6.5
AMNH A167570	F	46.4	3.2	5.6	3.8	2.8	2.1	3.0	15.7	19.3	14.1	8.1	23.6	24.1	23.9	11.0	8.0	3.0	7.2	1.8	6.2	7.0
AMNH A167576	F	50.6	3.4	6.1	3.8	2.6	2.6	3.5	16.2	22.3	14.1	9.1	25.1	24.0	23.7	10.7	8.3	3.2	8.5	2.1	6.0	6.3
UADBA 8231	F	47.6	3.3	6.3	3.8	2.5	2.4	3.9	15.8	19.0	14.4	12.4	24.3	24.0	23.6	12.0	8.3	2.7	7.3	1.8	5.9	7.1
AMNH A167596*[b]	SA	40.7	-	-	-	-	-	-	-	-	-	-	-	-	-	-	-	-	-	-	-	-
UADBA 8375	F	46.6	3.9	5.8	4.3	2.7	2.3	4.5	15.8	19.4	15.2	10.4	27.4	26.7	25.0	11.2	8.0	2.5	6.5	1.8	5.7	6.5
UADBA 8381	F	48.6	4.4	7.4	5.0	2.2	1.7	4.6	16.7	19.1	14.0	9.8	25.4	25.0	22.4	9.7	6.7	2.3	5.8	1.8	4.7	5.9
UADBA 8383	F	49.3	3.8	6.8	4.5	3.0	3.2	4.9	17.3	20.5	14.5	10.0	25.3	25.0	23.1	12.5	8.2	3.0	6.7	2.3	6.1	7.1
UADBA 8384	F	48.0	4.0	6.0	4.3	2.7	3.1	5.2	16.3	20.2	15.9	9.4	25.4	23.0	24.7	9.3	8.6	2.9	7.4	2.0	5.8	6.7
UADBA 8388	F	49.4	3.6	6.7	4.7	2.6	3.3	4.8	17.1	20.3	15.3	10.0	27.5	24.2	25.8	9.2	8.5	2.6	7.0	2.1	5.8	7.6
AMNH A167517	M	34.5	4.3	6.3	2.8	1.9	2.1	3.1	12.3	15.7	11.5	5.5	17.9	16.8	18.6	8.4	6.3	2.4	5.7	1.3	4.8	5.4
AMNH A167528	M	42.8	4.6	5.0	2.9	2.4	2.9	3.5	12.8	20.0	12.4	6.8	21.4	20.4	20.6	9.6	7.5	3.0	6.2	1.7	5.0	6.1
AMNH A167555	M	35.8	4.4	5.5	2.4	2.1	2.3	3.0	12.0	16.6	10.4	6.6	18.5	17.4	17.6	7.1	5.6	2.2	5.3	1.2	4.3	4.9
AMNH A167557	M	38.4	4.4	4.7	3.0	2.4	2.6	3.4	13.2	18.4	11.6	6.4	18.7	18.6	20.0	8.7	6.6	2.3	5.7	2.2	5.1	5.4
AMNH A167560	M	36.0	4.4	4.7	3.1	2.2	2.1	3.0	12.2	17.6	12.4	6.5	20.1	18.1	20.1	8.7	6.0	2.6	5.6	1.5	5.1	5.4
AMNH A167567	M	34.0	3.5	4.2	2.6	2.0	2.0	2.6	11.3	15.8	10.1	6.1	18.8	17.7	17.3	7.8	5.8	2.1	5.1	1.1	3.8	4.7
AMNH A167568	M	36.4	5.2	5.1	2.4	2.1	2.2	2.8	12.6	17.9	12.1	6.4	18.9	17.1	18.8	7.2	6.1	2.1	6.2	1.0	4.1	5.1
AMNH A167571	M	38.3	5.3	4.1	3.1	2.5	2.6	3.3	11.8	18.4	11.1	7.3	19.7	18.8	19.3	7.5	6.2	2.4	5.9	1.4	4.5	4.6
AMNH A167572	M	38.0	5.0	4.3	3.0	2.0	2.3	3.3	12.5	17.0	12.2	7.4	19.6	18.3	19.0	8.5	6.2	2.4	5.9	1.4	5.1	5.5
AMNH A167573	M	38.2	4.6	5.0	2.8	1.9	2.1	3.5	12.1	18.6	11.1	6.4	19.8	18.3	17.6	7.6	6.5	2.3	5.8	1.2	4.4	5.7
AMNH A167574	M	36.3	4.6	4.6	2.5	2.1	2.5	3.1	11.4	17.6	11.0	7.1	19.4	17.8	18.5	8.1	6.1	2.2	6.1	1.4	4.4	5.2
UADBA 8387	M	35.6	4.8	5.0	3.8	1.8	2.4	3.3	12.8	16.0	11.1	6.1	17.0	18.7	18.7	8.9	6.1	1.8	5.3	1.1	4.8	5.7
UADBA 8389	M	38.8	5.6	6.1	4.0	1.8	2.5	4.0	13.9	17.5	11.8	7.3	18.2	18.3	18.8	7.7	6.7	2.4	5.8	2.0	4.8	5.7
UADBA 8390	M	39.6	5.6	5.4	3.7	1.8	2.5	4.9	14.4	17.8	12.0	7.5	19.5	17.6	20.6	8.1	7.4	2.4	6.0	1.7	4.6	5.8
UADBA 8392	M	33.6	3.4	4.5	2.8	2.0	2.3	3.1	11.5	14.2	10.4	6.6	17.4	16.0	16.4	8.1	5.7	2.1	4.7	1.5	4.4	4.8
UADBA 8404	M	38.3	5.4	5.1	4.2	1.8	2.7	3.3	13.4	18.3	11.0	7.6	16.5	17.3	17.8	9.0	7.2	2.1	5.4	1.6	4.0	4.9

UADBA 8407	M	35.6	4.5	5.0	2.9	1.7	2.8	3.4	13.5	16.1	11.4	7.7	20.2	19.8	19.4	9.1	6.7	2.1	5.3	1.5	4.8	5.6

[b]ID Molecular

* Holotype

Table S12. Measurement data for *Mantidactylus tavaratra* n. sp.. All measurements in mm. F: female, M: male, J: juvenile, SA: subadult

Specimen	Sexes	SVL	TD	ED	EN	EST	NS	NN	HW	HL	HDL	RC	FE	TI	FT	TA	EO	EM	EHEAD	T1	TO3	TO5
AMNH A167507	F	52.0	3.5	6.1	3.8	3.0	3.1	4.6	17.9	22.6	15.8	9.8	28.0	27.8	27.0	13.0	5.9	3.6	7.5	2.9	6.8	7.5
AMNH A167508	F	55.6	4.0	6.5	4.2	2.8	3.2	5.1	19.0	24.0	15.6	10.2	27.5	26.5	25.9	12.6	10.0	3.5	8.0	1.8	6.3	7.0
AMNH A167510[b]	F	54.9	3.9	6.6	4.3	2.4	3.2	4.5	17.5	23.5	16.8	9.5	28.5	28.0	28.1	11.1	9.0	3.5	7.4	2.3	6.9	7.6
AMNH A167512	F	48.0	3.7	5.7	3.8	2.2	2.6	4.6	17.3	21.5	15.3	8.8	26.9	26.6	26.1	11.4	8.7	3.1	6.2	2.0	7.0	8.1
AMNH A167518	F	44.8	3.8	4.7	3.1	2.2	2.6	5.2	15.3	19.6	13.1	7.5	23.8	23.1	23.2	11.0	7.8	3.6	6.1	2.2	5.5	5.8
AMNH A167519	F	58.6	4.1	6.4	4.0	3.0	3.8	4.8	20.3	24.6	16.4	9.2	28.8	27.9	29.1	12.2	10.2	3.2	9.5	2.2	6.8	7.8
AMNH A167526	F	54.7	3.2	6.7	3.9	2.7	3.0	4.6	18.1	23.0	13.4	8.3	25.1	26.1	23.1	11.1	9.2	2.5	8.4	2.2	5.9	7.4
AMNH A167529	F	54.2	3.1	7.1	4.0	2.8	3.2	5.0	19.0	24.0	16.1	9.2	28.3	27.7	28.0	11.5	9.6	3.6	8.3	2.0	6.6	7.3
AMNH A167533	F	53.6	3.9	6.0	3.9	1.8	2.3	4.2	18.6	18.6	16.4	10.6	30.0	29.7	29.4	12.9	9.3	3.4	7.2	2.7	8.0	8.8
UADBA8117	F	61.0	4.0	7.7	4.4	2.8	3.6	5.9	21.3	26.1	18.4	12.0	34.3	31.7	33.4	13.7	10.8	3.5	8.8	2.9	9.9	10.6
UADBA8120	F	63.3	5.0	7.8	5.4	3.0	4.1	6.6	23.5	28.2	17.3	12.3	33.2	33.6	34.3	14.6	11.5	3.3	10.0	2.6	9.5	9.9
UADBA12155	F	59.2	4.8	6.6	4.7	2.2	2.4	4.7	19.0	25.3	16.0	9.8	27.3	26.3	27.8	11.7	10.1	3.4	7.8	2.2	7.0	7.8
UADBA12156	F	56.6	4.3	6.4	4.7	2.1	2.8	5.0	18.3	24.4	16.2	10.6	28.7	26.8	28.8	12.3	9.4	3.3	7.5	1.8	7.8	8.9
UADBA19619	F	54.1	3.8	6.7	4.1	2.8	3.2	4.7	18.7	24.2	14.6	9.5	27.8	27.4	27.3	12.5	8.8	3.2	8.3	2.4	6.6	7.4
UADBA19621	F	55.9	4.2	6.6	4.2	1.6	1.9	4.9	18.6	22.8	16.0	9.1	25.4	25.5	25.1	12.6	9.0	3.1	9.3	2.4	7.3	7.9
UADBA19622	F	56.3	4.3	7.4	4.1	2.7	2.3	4.9	18.0	23.7	15.5	9.6	27.2	26.7	26.9	12.4	9.0	3.4	8.0	2.0	7.2	8.3
UADBA19623	F	42.7	3.0	5.2	3.2	1.6	2.8	3.9	15.4	18.1	13.8	8.1	24.5	24.0	23.3	10.0	6.4	2.5	6.2	2.2	5.8	6.1
UADBA19624	F	51.9	3.3	5.9	3.7	2.3	2.7	4.8	17.5	22.4	15.1	9.0	26.6	27.4	26.7	11.7	8.0	3.1	7.6	2.2	6.8	7.7
UADBA19628	F	57.1	4.3	6.7	4.9	2.4	2.8	4.8	19.8	25.5	17.1	10.0	28.7	28.7	29.3	12.7	9.3	3.4	8.3	2.6	7.6	8.6
UADBA19629	F	53.8	3.9	5.9	4.2	2.9	2.1	4.7	19.8	23.2	16.3	9.2	27.2	27.4	27.5	12.9	8.4	3.2	7.1	2.1	6.7	8.5
UADBA19633	F	53.6	3.6	6.3	4.5	2.4	3.3	4.3	17.9	22.6	15.1	9.5	27.1	27.4	25.8	12.3	8.8	3.1	7.9	2.2	7.0	8.3
UADBA19634	F	56.2	3.8	6.6	4.2	2.6	3.1	4.9	18.4	24.8	15.5	9.6	28.0	27.3	26.5	11.8	9.1	3.4	8.4	2.3	6.7	8.3
UADBA19636	F	55.1	4.2	6.6	4.5	2.6	3.7	5.4	19.4	23.6	15.6	9.0	26.7	26.5	26.4	11.7	9.7	3.2	8.0	2.4	7.2	7.7
UADBA19637	F	57.7	4.5	7.1	4.3	2.6	3.0	5.1	19.2	24.8	16.6	9.2	28.4	28.0	28.2	12.4	9.7	3.4	8.5	2.6	7.9	8.0
UADBA19638	F	49.3	3.2	5.6	4.3	2.5	2.8	4.9	17.3	21.8	14.4	8.8	26.1	26.6	25.1	11.0	8.2	2.9	6.6	2.4	7.1	7.7
UADBA19639	F	57.6	4.0	6.4	3.8	2.2	3.4	5.3	19.2	25.2	17.0	8.8	26.7	27.8	26.1	12.4	8.9	3.6	9.2	2.5	7.3	8.4
UADBA 19650	F	54.0	3.0	6.6	4.3	2.1	1.9	4.5	17.6	22.0	14.8	10.7	27.8	27.5	26.0	11.6	9.9	3.4	8.2	2.5	7.2	7.8

UADBA26108	F	58.3	3.7	5.6	4.3	3.0	3.0	4.6	19.6	24.8	15.2	10.0	25.2	27.3	26.7	11.6	10.3	3.0	8.7	2.6	7.1	8.0
UADBA26109	F	53.5	3.7	7.1	4.3	2.7	4.0	5.4	18.2	21.7	17.1	9.8	26.4	26.5	26.5	12.1	8.7	3.3	8.2	1.8	6.0	6.8
UADBA26110	F	55.4	3.8	6.5	4.6	2.6	3.0	6.0	18.8	23.8	16.6	10.0	28.3	29.0	27.4	13.8	8.9	3.1	7.5	2.4	6.5	8.0
UADBA26310	F	51.9	4.0	6.5	4.7	2.4	3.6	5.9	20.0	24.7	18.3	10.6	28.7	29.0	30.5	12.8	8.5	3.5	8.0	2.3	7.5	8.7
UADBA26311	F	52.6	4.2	6.7	3.4	2.4	3.4	4.9	18.2	23.3	12.1	9.6	28.4	27.0	28.7	12.0	8.6	3.1	8.6	2.5	7.4	8.3
UADBA26312	F	56.2	4.7	7.6	4.7	2.2	4.4	5.9	20.6	23.0	17.0	9.9	28.2	27.5	27.2	12.1	9.1	3.6	9.0	2.5	5.9	6.5
UADBA26313	F	52.8	3.8	6.1	4.3	2.7	3.4	4.8	18.3	24.3	16.7	9.7	29.0	28.4	27.7	12.9	8.6	3.1	8.4	2.4	6.8	7.2
UADBA26314	F	48.4	3.7	6.0	4.4	2.4	3.5	5.0	16.6	22.2	15.7	8.8	26.9	28.2	26.0	13.0	7.8	3.0	6.8	2.2	6.6	7.2
UADBA26315	F	61.4	4.4	7.7	4.7	2.4	3.5	5.0	20.2	24.7	17.6	8.7	30.7	30.1	28.4	13.0	9.6	3.4	8.8	2.4	6.4	7.1
UADBA26316	F	61.4	4.3	7.4	4.7	2.2	3.8	6.6	19.7	26.6	17.2	10.3	27.0	28.2	27.0	11.7	10.1	4.0	8.9	2.2	6.1	7.4
UADBA26317	F	52.6	3.8	6.6	4.9	2.7	3.1	4.8	18.0	22.6	15.2	9.7	28.0	28.6	26.6	12.1	8.5	3.4	8.2	2.1	6.6	7.7
UADBA26318	F	49.1	3.4	5.7	4.2	2.4	3.5	4.7	16.4	21.4	16.6	9.0	27.4	28.4	27.6	12.5	9.3	3.6	8.0	2.5	7.5	8.2
UADBA26319	F	57.6	4.2	6.4	5.1	2.1	3.4	4.8	18.5	24.8	15.7	9.3	27.1	27.3	27.8	13.2	9.6	3.1	8.1	2.3	6.7	7.9
UADBA26320	F	52.1	3.9	6.6	4.4	3.0	3.1	5.5	18.0	23.5	15.8	8.7	26.1	25.5	26.4	12.2	8.6	3.2	9.0	2.1	6.6	6.9
UADBA26321	F	57.6	4.3	6.6	4.8	2.4	2.5	5.4	18.5	23.6	15.7	10.4	28.6	28.1	28.0	14.0	9.4	3.6	7.4	2.5	7.3	7.8
UADBA26322	F	56.7	4.3	6.0	4.4	2.7	2.8	4.7	19.7	25.5	16.5	9.1	26.8	27.9	28.4	12.4	9.2	3.2	8.6	2.0	7.5	8.5
UADBA26323	F	56.0	4.2	6.7	4.8	2.5	3.5	4.8	18.6	23.6	17.1	9.0	29.2	27.4	29.1	12.3	9.4	3.0	8.6	2.5	7.6	8.1
UADBA26324	F	53.8	3.8	6.5	4.1	3.1	3.1	5.4	18.4	22.3	16.8	9.7	28.7	28.4	28.5	13.4	9.1	3.5	7.8	2.1	7.5	8.4
UADBA26325	F	60.6	4.2	6.6	4.6	2.6	3.3	6.3	20.6	25.6	17.6	11.0	31.6	30.6	29.8	13.5	9.3	3.2	9.1	2.5	7.3	8.2
UADBA26326	F	52.4	3.1	5.4	3.2	2.6	2.4	3.6	18.0	23.3	14.4	8.3	25.1	25.2	24.4	11.4	7.9	2.9	7.6	1.7	6.8	7.0
UADBA26328	F	58.4	4.0	7.2	5.0	2.7	4.0	5.0	20.0	24.2	17.1	11.7	30.4	30.0	29.2	13.2	9.5	3.6	8.6	2.8	7.1	7.5
UADBA26330	F	56.7	4.5	7.0	4.9	1.4	3.3	5.4	19.0	24.4	17.5	9.7	29.5	30.4	29.0	14.6	8.9	3.3	7.4	2.1	7.2	8.1
UADBA26331	F	58.1	4.0	7.7	4.4	2.5	4.0	6.5	19.4	24.8	18.2	10.0	27.1	30.0	28.0	14.0	9.9	3.4	8.9	2.6	7.2	7.9
UADBA26332	F	53.0	3.1	5.2	4.3	2.1	3.1	4.3	17.5	22.8	14.3	8.7	26.8	27.0	26.1	12.0	9.0	3.1	7.9	2.1	6.8	8.2
UADBA26334	F	49.4	3.8	6.6	4.2	2.1	3.4	4.6	17.6	22.8	15.2	8.6	27.1	27.8	25.3	11.3	8.8	3.3	7.7	2.4	5.9	8.1
UADBA26335	F	50.5	3.2	5.8	4.2	2.3	2.1	4.6	17.6	21.0	10.8	7.8	24.0	26.0	26.5	10.9	8.1	2.9	7.4	2.2	6.7	7.9
UADBA26336	F	56.1	3.9	6.3	4.3	2.8	4.0	5.0	19.5	20.7	15.2	9.5	28.3	27.5	25.6	12.6	8.6	3.3	7.7	2.4	6.2	7.8
UADBA26338	F	53.8	4.4	6.1	4.0	2.3	3.1	5.6	17.3	24.0	16.4	8.7	26.7	27.2	28.0	13.3	6.5	2.5	6.3	1.7	6.6	7.5
UADBA26340	F	57.6	4.4	7.7	4.4	2.7	3.4	5.1	19.3	25.2	17.6	10.0	29.0	28.2	28.5	13.2	8.6	3.1	7.8	2.1	7.7	8.6
UADBA26341	F	50.0	3.9	6.2	4.3	2.4	3.6	5.8	18.3	22.6	15.3	9.4	29.6	27.8	26.8	12.0	8.9	3.4	8.0	2.2	6.8	7.2
UADBA26393	F	53.8	2.6	6.0	3.5	2.0	2.5	4.3	18.0	22.6	14.8	8.4	27.3	26.3	26.0	11.6	9.0	3.3	9.3	2.2	6.5	7.9
UADBA26394	F	57.7	3.3	6.2	4.5	2.5	3.1	4.6	19.0	22.7	16.4	8.8	29.4	28.4	28.6	12.5	9.6	2.6	8.8	2.5	7.2	8.3
UMMZ 212833	F	53.7	4.0	6.9	4.0	0.8	2.0	4.1	19.2	21.0	14.7	9.9	27.0	26.3	25.3	12.1	10.2	4.3	8.4	2.8	5.7	6.7

AMNH A167513	F	47.5	3.2	5.6	3.8	1.9	2.5	3.7	15.7	19.5	14.2	8.3	25.7	26.0	25.4	11.3	8.0	2.8	6.5	1.4	5.8	6.5
AMNH A167515[b]	M	45.3	4.5	5.0	3.5	2.2	2.4	4.2	15.3	21.4	14.4	7.5	23.3	22.5	23.3	10.4	8.1	3.5	7.6	1.8	4.9	6.7
AMNH A167520	M	48.8	4.7	5.4	3.7	2.3	2.4	3.9	15.8	21.6	15.6	7.7	24.4	24.4	25.1	12.4	8.4	3.2	7.1	2.1	5.4	6.8
AMNH A167524[b]	M	41.1	4.6	5.2	3.1	2.1	2.3	4.0	12.0	19.5	12.6	6.8	21.2	20.3	21.2	9.0	7.5	2.6	6.5	2.2	5.2	5.8
AMNH A167531	M	38.6	3.1	4.2	2.7	1.5	2.2	3.4	12.9	17.3	11.6	7.1	21.4	19.9	19.6	9.2	6.5	2.5	5.3	1.7	4.6	5.3
AMNH A167532	M	37.0	3.0	4.9	2.7	1.5	2.7	3.4	12.4	16.4	10.8	6.2	21.6	20.2	19.8	8.7	6.2	2.2	4.5	1.2	4.7	5.1
UADBA 19625	M	39.9	4.5	4.8	3.6	1.8	2.3	3.3	13.9	19.4	11.7	7.1	18.7	19.3	20.1	9.4	6.4	2.2	5.7	1.5	5.0	5.1
UADBA 19630	M	43.3	5.3	5.3	3.5	1.8	1.7	4.0	15.4	19.4	12.7	7.4	21.2	21.4	21.2	10.0	8.1	2.8	7.4	2.1	7.2	7.8
UADBA 19644	M	35.7	5.1	4.6	4.5	1.7	2.1	3.5	11.6	16.2	11.9	7.0	18.7	18.6	20.2	8.2	6.0	1.8	5.4	2.0	4.5	5.0
UADBA 26306	M	38.5	4.0	4.9	3.6	1.7	2.8	4.2	13.7	17.6	13.2	7.6	20.3	21.7	21.4	10.8	6.3	2.0	5.3	1.6	5.0	5.1
UADBA 26308	M	43.6	5.1	6.0	4.1	2.0	2.6	4.3	15.1	19.5	13.7	7.5	22.1	22.8	22.7	9.8	7.0	2.6	6.2	2.1	5.8	6.2
UADBA 26309	M	44.4	5.2	5.2	4.2	2.4	3.2	4.4	15.4	20.5	14.6	8.1	22.6	22.1	23.5	10.2	6.6	2.4	6.0	1.6	5.3	5.4

[b]ID Molecular

* Holotype
